# Chemical Control of Cuprite Crystal Growth and Morphology toward High-Index Surfaces

**DOI:** 10.1021/acs.inorgchem.6c03779

**Published:** 2026-10-10

**Authors:** Gaia Novo, Falak Shafiq, Tatiana Rodriguez-Flores, Roberto Scotti, Massimo Moret, Roberto Nisticò

**Affiliations:** Department of Materials Science, 9305 University of Milano-Bicocca, U5, INSTM, Via R. Cozzi 55, 20125 Milano, Italy

## Abstract

The morphology of cuprite (Cu_2_O) crystals is governed by the interplay among reduction chemistry, solution composition, nucleation kinetics, and selective stabilization of crystallographic planes during growth. In this work, these factors were systematically investigated through two wet-chemical precipitation routes employing either glucose or a poly­(vinylpyrrolidone)/ascorbic acid (PVP/AA) system as reducing and crystal growth-directing agents. The glucose-mediated route showed a strong dependence on alkalinity and glucose concentration, producing a progressive evolution from octahedral to truncated octahedral, cubic, and complex polyhedral morphologies, exposing {100}, {110}, {111}, and high-index facets. In the PVP/AA system, decoupling the reducing and capping functions promoted the formation of rounded particles characterized by a dense arrangement of high-index facets, whereas octahedral crystals can also be obtained under specific conditions, demonstrating that the stabilization of {111} facets does not exclusively depend on glucose. Comparative experiments showed that PVP alone yielded CuO, whereas AA promoted Cu_2_O formation, highlighting the distinct roles of reduction and surface modification in the crystal growth. Overall, the results demonstrate that solution chemistry and selective surface interactions can be exploited to control Cu_2_O morphology and crystallographic facet exposure.

## Graphical abstract

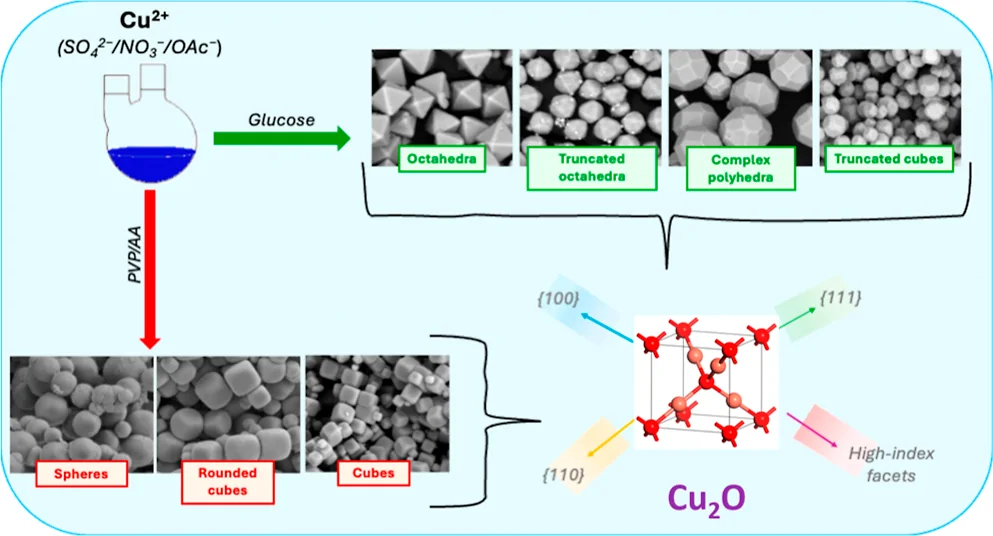

## 1 Introduction

Copper oxides, principally cuprous oxide (cuprite, Cu_2_O) and cupric oxide (tenorite, CuO),^1–3^ have sustained scientific interest across a broad spectrum of relevant applications, including catalysis,^4,5^ sensing,^6^ and electrochemical energy storage.^7^ Among these, the electrocatalytic CO_2_ conversion has emerged as a particularly compelling focus, driven by the urgent global imperative to reduce carbon emissions and generate valuable chemical feedstocks from renewable energy sources.^8,9^ In particular, both Cu_2_O and CuO are intrinsically active electrocatalysts by virtue of their accessible and interconvertible Cu(0)/Cu­(I)/Cu­(II) redox states, which directly modulate the surface electronic structure, intermediate binding energy, and product selectivity across a range of electrochemical reactions, including the CO_2_ reduction (e-CO_2_RR).^10–12^ In this regard, the electrocatalytic performance of these oxides is governed not by bulk composition alone but predominantly by the crystallographic identity of bounding facets, the density of catalytically active surface sites (partly determined by the former), and the binding energy of the reaction intermediates. Therefore, precise nanostructure control and facet engineering are fundamental requirements for achieving a targeted activity and selectivity in such electrocatalytic processes.^13,14^

Achieving deliberate control over the exposed facets during the synthesis process demands control over the nucleation kinetics (which sets the crystal structure with its pertinent chemical composition and crystallographic symmetry) and the subsequent anisotropic crystal growth (determined by the expansion of facets controlled by supersaturation, growth mechanisms, and the interfacial attachment energies of the growth units).^15–17^ Capping agents are deputized to regulate the nucleation steps (e.g., controlling polymorphism) and/or growth rates of different crystallographic planes. These additive molecules act through adsorption on surfaces or kink sites, depending on their chemical nature (e.g., small molecules or polymers, neutral or charged functional groups), mimicking the structure of the growing surfaces. Growth additives or impurities can exhibit a thermodynamic effect by reducing the step or surface energy (thus increasing crystal growth rates) or by blocking the growth sites (typically the kink sites) even at very low concentrations.^18^ In most cases, reduction of growth rate is observed, including the formation of a dead zone with surfaces not growing below a critical supersaturation value.^19^ Selectively reducing the growth rate of specific crystal faces provides a growth morphology largely dominated by the slowly growing surfaces. Therefore, tailoring of crystal growth morphologies is available to control the desired physicochemical properties.^20,21^

Concerning the Cu_2_O systems, the three principal low-index forms (note: a short glossary for crystallographic symbols and terms is included at the end of the Supporting Information), namely {100} (cube), {111} (octahedron), and {110} (rhombic dodecahedron), carry distinct surface energies and coordinative environments, and their relative morphological relevance is strongly influenced by the choice of both capping and reducing agents.^22^ Among capping agents, both nonionic [e.g., poly­(ethylene glycol) (PEG) and poly­(vinylpyrrolidone) (PVP)], and ionic [e.g., sodium dodecyl sulfate (SDS) and cetyltrimethylammonium bromide (CTAB)] surfactants interact with developing Cu_2_O crystalline surfaces primarily through the coordination of their polar/ionic groups with surface Cu atoms and/or through steric hindrance between hydrophilic polymer chains and the aqueous growth medium.^23^ The reducing agents [e.g., ascorbic acid (AA), sodium borohydride (NaBH_4_), and glucose], instead, perform a distinct but equally consequential stage: by controlling the rate of Cu­(II) to Cu­(I)/Cu(0) conversion, they govern the degree of supersaturation and, consequently, determine whether the crystal growth proceeds under either low or high supersaturation levels, which in turn activate different crystal growth mechanisms and the associated kinetic laws.^24–26^

In recent years, the synthesis of Cu_2_O crystals with tailored morphologies has received significant attention from the scientific community.^27–29^ Among various synthesis methods, wet chemical routes have been widely investigated/exploited to synthesize various metal oxide well-ordered structures, as they present several practical advantages, such as being simple, straightforward, scalable, and rapid (thus compatible with the industrial scaling up).^30^ In this context, various literature studies documented the roles of different capping agents on copper oxide architectures. For instance, Ho et al.^31^ demonstrated that performing a systematic variation of the concentration of the ionic surfactant SDS in hydroxylamine-mediated Cu_2_O synthesis drives toward a morphological evolution from cubic morphology through truncated octahedra to rhombic dodecahedra, thus establishing a direct and reproducible correlation between the concentration of the capping agent and the relative growth rates of different crystal planes. Chen et al.,^32^ instead, demonstrated that by working in a polyol environment constituted by PVP and diethylene glycol it is possible to obtain Cu_2_O materials with different architectures by simply varying the nature of the Cu salts’ counterion (i.e., acetylacetonate, hydroxide, and acetate), obtaining either cubic, flower-like, or hierarchical microsphere structures, respectively. The reason behind this trend is the selective adsorption/interaction of polyols on the distinct crystal facets of Cu_2_O by varying the operating conditions. Interestingly, Li et al.^33^ constructed CuO/Cu_2_O hybrid heteronanosheets bridged by interfacial Cu­(II)–O–Cu­(I) bonds via in situ topotactic partial reduction of CuO sheets using the reducing agent ascorbic acid (AA), selectively converting surface CuO domains into Cu_2_O ones. Lastly, Dourado et al.^34^ demonstrated that glucose (which acts both as a capping and reducing agent) can selectively adsorb on the Cu_2_O {100} surfaces, thus leading to the occurrence of specific morphologies. Furthermore, authors observed that the Cu_2_O cubic systems present {100} crystal facets with an excess of oxygen atoms on the surface, leading to negative electrostatic potentials, whereas the Cu_2_O systems presenting the {111} octahedral facets exhibited more positive surface potentials. This important result suggests that Cu_2_O cube faces interact less favorably with negatively charged ions (such as ascorbate species) than the octahedra, due to a higher repulsive electrostatic interaction with anions.^35^ However, despite the numerous studies reported in the literature, there is still clarity to be gained regarding the physicochemical rules behind the possibility of finely controlling the growth of well-ordered crystalline architectures. To quote the famous phrase of Feynman, “there’s plenty of room at the bottom”.^36^

Importantly, the electrocatalytic behavior of copper oxides in e-CO_2_RR is strongly influenced by their surface structure, as different crystallographic facets expose Cu atoms with distinct coordination environments and surface energies. Experimental and theoretical studies have demonstrated that Cu_2_O crystals exposing different low- and high-index facets exhibit distinct interactions with CO_2_ reduction intermediates, resulting in variations in catalytic activity and product distribution. In particular, Cu_2_O crystals dominated by low-index {100} and {111} facets, as well as those exposing high-index surfaces, have been reported to display different e-CO_2_RR performances, reflecting differences in surface atomic configurations and the associated adsorption properties. High-index facets, characterized by a higher density of stepped, kinked, and under-coordinated Cu sites, can provide additional reactive sites for catalytic transformations. Notably, however, the initial Cu_2_O surface is not necessarily preserved under electrochemical operating conditions, and facet-dependent surface reconstruction can substantially influence the resulting catalytic behavior. Therefore, controlling the crystal habit and the selective exposure of specific crystallographic planes represents an effective strategy not only for understanding crystal growth mechanisms but also for designing oxide-derived Cu-based catalysts with tailored surface properties.^37–44^

Despite these advances, achieving precise control over copper oxide morphology and high-index surface exposure through solution-phase synthesis remains challenging. Understanding how solution chemistry regulates nucleation and crystal growth is hence essential for the rational preparation of cuprite crystals with controlled surface structures and, ultimately, for establishing well-defined structure–property relationships in Cu-based functional materials.

Therefore, in the present study, we systematically explored the synthesis of Cu_2_O particles following simple precipitation routes. A comparison between two different capping systems, namely, a single-component glucose-mediated route (i.e., glucose acts as both a capping and reducing agent) and a bicomponent PVP/AA-mediated route (i.e., PVP acts separately as a capping agent, whereas AA acts as a reducing agent and growth additive), has been performed. The final objective of this study is to understand the role of the different experimental parameters for each synthesis route in driving the growth toward the formation of specific crystal facets, thus, in other words, unveiling the mechanistic role of every ingredient/parameter in driving/tuning the synthesis toward specific architectures. To do so, multiple synthesis conditions (i.e., reaction volume, synthesis temperature and time, concentration of capping/reducing agents, and selection of Cu precursors) are explored, and relevant considerations are highlighted. The mechanistic interpretation proposed in the present study is based on experimentally observed morphology trends and classical concepts of crystal growth, providing a qualitative description of the factors governing observed morphological evolution.

## 2 Results and Discussion

### 2.1 Morphology Influence on Cuprite Induced by Glucose

The following paragraph focuses on the investigation of the effects of compositional and experimental parameters on the final morphology of Cu oxides produced by employing glucose as the only capping/reducing agent and NaOH as the precipitating agent.

#### 2.1.1 Order and Delivery Method of Reactants

A change in the order and the delivery method of reactants can significantly impact the crystals’ formation and growth.^45^ For both Glu-1 and Glu-2 samples, the required amount of NaOH was added directly as solid pellets and allowed to dissolve in the reaction mixture, thus no initial NaOH solution concentration can be defined for these experiments. In contrast, the Glu-3 sample employed a preprepared 12 M NaOH solution added dropwise to investigate the effect of the hydroxide delivery method. Figure 1 reports the SEM micrographs of particles obtained by varying both the reactants’ order and delivery methods, whereas the relative XRPD analysis is reported in the Supporting Information (Figure S1). As shown in Figure 1a, when the precipitating agent NaOH was delivered in powdery form prior to the capping/reducing agent glucose (Glu-1 sample), the final growth habit appeared octahedral, with an average size of ca. 3.57 ± 0.43 μm. The sample was characterized by XRPD, showing the presence of the main crystal reflection planes attributable to the predominant cuprite (Cu_2_O) crystal phase at 2θ = 29.6° (110), 36.4° (111), 42.3° (200), 52.5° (211), 61.4° (220), 73.6° (311), and 77.4° (222) (Cu_2_O, 01–078–2076), and the presence of a minor extra signal at 2θ = ca. 43.3° (111), attributable to the presence of traces of metallic Cu (Cu, 01–085–1326; Figure S1a). Importantly, although pronounced changes in crystal habit are observed by SEM analysis (vide infra), the relative intensities of the XRPD reflections remain broadly comparable with those of the reference powder pattern of the cubic Cu_2_O crystal phase. This is consistent with the predominantly random orientation of the largely equant particles forming the powdery samples. Thus, the combination of XRPD and SEM provides complementary information: XRPD establishes the phase and crystal structure, whereas SEM allows the evolution of the external crystal habit to be directly assessed.

**Figure 1:**
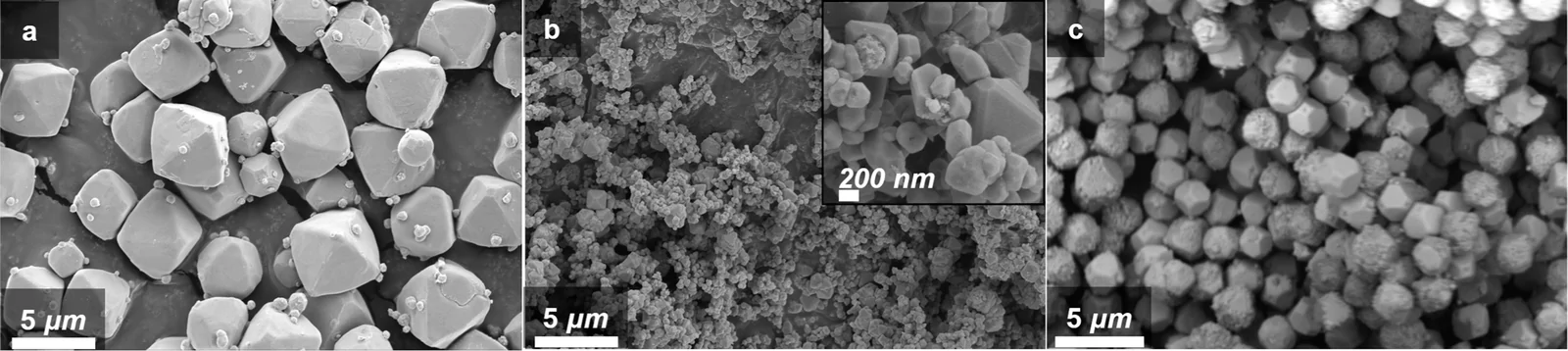
SEM micrographs of Glu-samples: effect of delivery and order or reactants, namely: (a) Glu-1 (NaOH in pellets; NaOH, Glu), (b) Glu-2 (NaOH in pellets; Glu, NaOH), and (c) Glu-3 (addition of a 12 M NaOH solution; NaOH, Glu). All micrographs were collected at the same magnification (10k ×), whereas the inset in panel (b) was collected at high magnifications (50k ×).

By changing the order of reactants (by adding first the capping/reducing agent glucose and then the precipitating agent NaOH), a loss of control over the morphology was registered, obtaining small (ca. 443 ± 234 nm) particles with a part of them being euhedral and a part being irregular anhedral lacking any distinct polyhedral shape (Glu-2 sample; Figure 1b). The XRPD diffractogram of the Glu-2 sample shows the main signals correspond to the Cu_2_O crystal phase, coupled with the presence of two minor extra signals, the first one at ca. 38.8°2θ (111) attributable to traces of tenorite (CuO) crystal phase (CuO, 01-080-1268), and the second one at ca. 43.3°2θ (111), attributable to traces of metallic Cu (Cu, 01-085-1326; Figure S1b). Experimental evidence clearly demonstrated that reversing the addition order between NaOH and glucose significantly affects the synthesis outcome, leading to different phase compositions and crystal morphologies. In fact, in the first case (Glu-1), the addition of the precipitating agent NaOH to the Cu^2+^ solution resulted in the precipitation of black tenorite CuO, identified as the predominant Cu-containing phase. After the subsequent addition of glucose, the CuO phase was converted into cuprite Cu_2_O, indicating the occurrence of a glucose-mediated reduction process of Cu­(II)-containing species under the investigated conditions. Vice versa, in the second case (Glu-2), glucose addition to the Cu^2+^ solution did not induce visible precipitation, suggesting that Cu^2+^ species remained stabilized in solution before alkalinization. After the subsequent addition of NaOH, a gradual color change from a blue solution toward the formation of orange precipitates was observed, associated with the formation of Cu_2_O precipitates. The smaller average particle size observed for the Glu-2 sample suggests a higher nucleation during the early stages of crystal formation compared with the Glu-1 sample, resulting in the generation of a larger number of smaller crystals. Although the effective supersaturation cannot be directly quantified in the present reaction system because it continuously evolves during the Cu­(II) hydrolysis, glucose oxidation, Cu­(II) reduction, and Cu_2_O precipitation (i.e., Cu_2_O does not exhibit congruent solubility), the experimental variables investigated in this work are expected to modify the supersaturation profile by influencing the solution chemistry. Accordingly, the following discussion interprets supersaturation as a mechanistic concept rather than as a directly measurable quantitative parameter. In this context, the higher effective supersaturation in the Glu-2 synthesis is attributed to glucose reducing the Cu^2+^ ions released upon dissolution of the soluble salt, whereas sample Glu-1 exploits the equilibrium Cu^2+^ concentration established by the CuO precipitate and/or soluble tetrahydroxocopper­(II) species arising from excess NaOH. These synthesis processes are very different, and so it is not surprising that they lead to the formation of particles of completely different sizes and morphologies. Moreover, by simply changing the delivery method of the precipitating agent (NaOH) from a powdery form (Glu-1) to a dropwise addition of a 12 M NaOH solution (Glu-3), but maintaining the order of addition of Glu-1 sample (i.e., NaOH prior to glucose), the formation of particles with a cube-truncated octahedral morphology was observed, presenting an average particle size of ca. 2.04 ± 0.29 μm (Glu-3; Figure 1c). The structural analysis revealed the occurrence of Cu_2_O phase with traces of CuO (a minor signal at ca. 38.8°2θ, Figure S1c). In this context, even the precipitating agent (NaOH) delivery method seems to affect both particles’ morphology and size. The main reason behind this phenomenon seems attributable to the different pH changes, as in the Glu-1 sample, the solution pH change is gradual since NaOH pellets take ca. 5 min to completely dissolve, whereas in the Glu-3 sample the NaOH solution was added directly to the solution, thus causing a more rapid pH change, even if during the experiment the delivery rate was maintained comparable to the pellets’ dissolution rate.

In this regard, it should be noted that although the initial hydroxide concentration was precisely defined by the experimental formulation, the solution pH was not monitored continuously during the reaction. Therefore, the present discussion refers to controlled initial reaction conditions.

#### 2.1.2 Initial Volume of Deionized Water

Another side effect correlated to the pH change is the effect of concentration obtained by varying the volume of the reaction (i.e., maintaining constant the molar ratio of all ingredients). Figure 2 reports the SEM micrographs of micrometer-sized particles obtained by varying the initial volume of deionized water, whereas the relative XRPD analysis is reported in the Supporting Information (Figure S2). In particular, the water volume varied as follows, namely: 80 mL (Glu-4; Figure 2a), 100 mL (Glu-1; Figure 2b), 120 mL (Glu-5; Figure 2c), and 200 mL (Glu-6; Figure 2d). The morphological analysis revealed that the change in deionized water volume significantly affects the morphology of the particles, registering a gradual transition from octahedra with straight edges (Glu-4, ca. 3.02 ± 0.44 μm), octahedra with increasingly curved edges (Glu-1 and Glu-5, ca. 3.57 ± 0.43 μm and ca. 2.96 ± 0.37 μm, respectively) to octahedra truncated by cube and rhombic dodecahedron faces (Glu-6, ca. 3.18 ± 1.52 μm) by increasing the volume, thus indicating a tendency toward the formation of more equant particles. In addition to the dominant micrometer-sized octahedral particles, smaller particles can be observed decorating the surfaces of the larger crystals in some samples, particularly Glu-4 (Figure 2a) and Glu-1 (Figure 2b). These secondary particles may originate from secondary nucleation occurring during the later stages of the reaction, when residual Cu-containing species remain available in solution after the formation of the primary crystals. Alternatively, their formation may be associated with surface oxidation and subsequent reprecipitation processes. However, the available experimental evidence does not allow these possible origins to be unambiguously distinguished. Their density and spatial distribution were not quantitatively evaluated, also because the population of these small particles may evolve upon sample aging.

**Figure 2:**
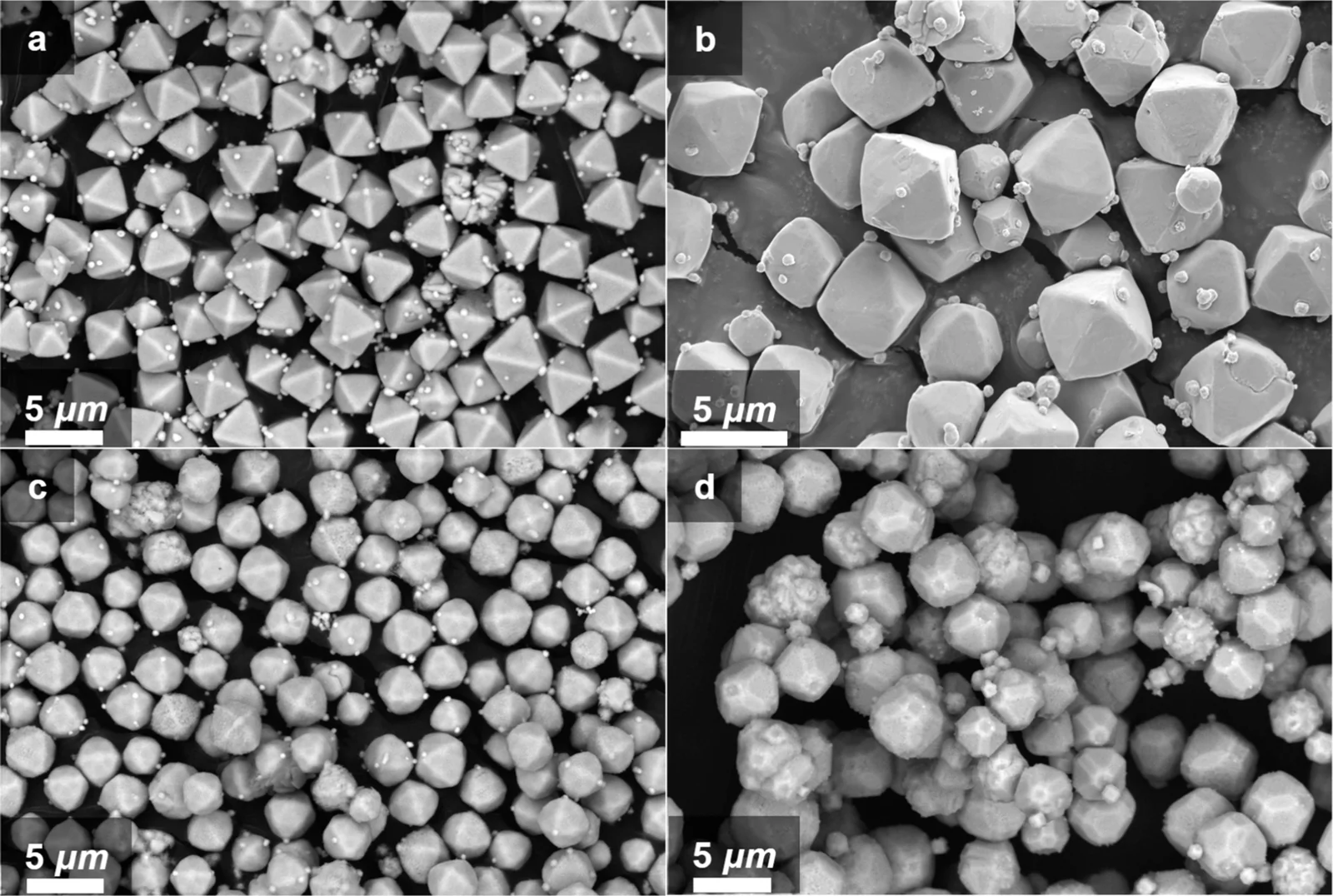
SEM micrographs of Glu-samples: effect of the initial volume of deionized water, namely: (a) Glu-4 (80 mL), (b) Glu-1 (100 mL), (c) Glu-5 (120 mL), and (d) Glu-6 (200 mL). All micrographs were collected at the same magnification (10k ×).

Regarding the dominant crystals, the effect of the hydroxide concentration should be interpreted in terms of the changes it induces in solution chemistry, including copper speciation and precipitation kinetics, which are expected to modify the effective supersaturation profile during crystal formation. Consequently, the balance between nucleation and facet-dependent crystal growth changes, leading to different equilibrium morphologies. Starting with Glu-1, the crystal morphology evolves from neat octahedra (with only traces of rounding) in Figure 2a to increasingly more evident loss of flatness of the octahedra (Figure 2b,c) with small cube truncations. These strange morphologies can be explained with the presence of faces with higher Miller indexes. Cuprite is well known for its huge number of observed crystal forms, at least 20 of them recognized in mineralogical samples.^46^ In the classical Atlas der Kristallformen,^47^ some hundred cuprite crystals are reported, showing the variety of crystal morphologies given by variations of the cube, octahedron, and rhombic dodecahedron mixed with high-index surfaces (see Figure S3). The polyhedra in Figure 3a,b show how simple it is to modify regular octahedra with just one additional family of high-index planes to approach morphologies observed in Figure 2b,c. The polyhedron in the Atlas der Kristallformen (Image 26, Table 140, Volume 7; Figure S4)^47^ closely resembles Figure 3a built with the six {111} faces and 24 facets belonging to the {320} form. Introducing a form {321} with multiplicity^48^ (Figure 3b), one can model a smoother octahedron. Figure 3c shows that only certain forms can satisfy the appearance of our SEM images or the quoted Atlas der Kristallformen^47^ because the apical regions along the crystallographic axes are too flat with {310}. On further increasing the reaction volume, the morphologies reported in Figure 2d exhibit more complex morphologies with {100}, {110}, {111} plus {211} facets (Figure 3d). It also emerges from these preliminary observations that the stabilization of a few high-index facets can lead to rounded crystals. In the following paragraphs, a structural justification of this rationale is proposed while discussing other complex morphologies observed for cuprite microcrystals. Moreover, the proposed presence of {32l} high-index facets represents a crystallographic model consistent with the observed morphology, successfully reproducing the progressive rounding of the octahedral edges and closely resembling morphologies reported for natural cuprite crystals. Although the present analysis does not uniquely identify the Miller indices of the exposed high-index facets, it provides a plausible crystallographic interpretation of the observed crystal habits.

**Figure 3:**
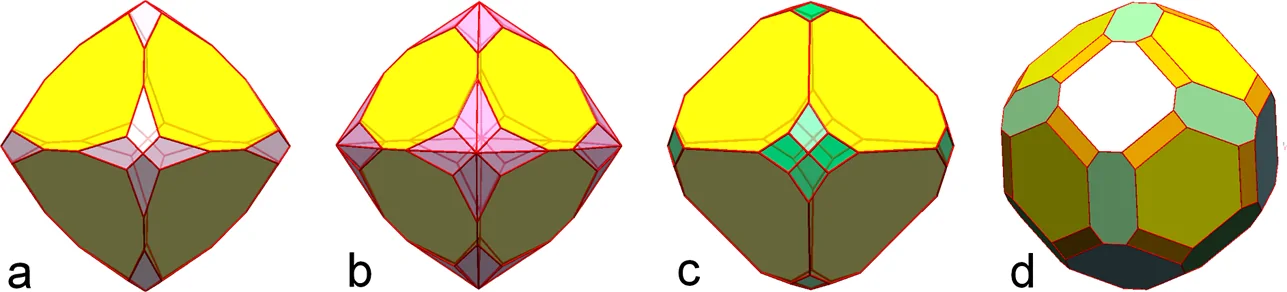
Crystal morphologies obtained by introducing to an octahedral crystal characterized by {111} faces (yellow): (a) {320} faces (light violet), (b) {321} faces (violet); (c) {310} faces (green), and (d) {110} (light green) and {211} (orange) faces.

The morphological analysis carried out on the cuprite samples in Figure 2 highlighted that by doubling the volume of deionized water, dilution modifies the concentration of reactive copper species, pH and the precipitation kinetics, thereby changing the effective supersaturation profile and favoring the development of {100} and {110} truncated octahedral morphology. This result further strengthens the previously made hypothesis regarding the correlation between the NaOH concentration and the appearance of the {110} surfaces (as evidenced in the Glu-3 sample). XRPD analysis confirmed the occurrence of the Cu_2_O crystal phase for all samples, with traces of metallic Cu in the case of both Glu-4 and Glu-1 octahedral samples obtained at higher concentrations and traces of CuO crystal phases in the case of both Glu-5 and Glu-6 samples obtained at high dilution (Figure S2). Furthermore, it is not possible to recognize a clear variation in the average particle size associated with the dilution phenomena induced by the volume variation (see Table 1).

**Table 1:** Compositional and Experimental Parameters Adopted during the Synthesis and Structural and Morphology Characterization of Glu-Samples, Namely by Employing Glucose as a Capping/Reducing Agent^a^

samples	Cu(II) salt	NaOH (g)	Glu (g)	water volume (mL)	temperature (°C)	delivery and order of reactants	reaction time (min)	XRPD			SEM	
								crystal phase	Cu_2_O lattice parameters *a* = *b* = *c* (Å)	Cu_2_O Scherrer crystallite average size τ (nm)	morphology	average particle size (nm)
Glu-1	sulfate	9.6	1.8	100	70	NaOH in pellets. NaOH, Glu.	10	Cu_2_O (Cu)	4.27	68	octahedral	3573 ± 432
Glu-2	sulfate	9.6	1.8	100	70	NaOH in pellets. Glu, NaOH.	10	Cu_2_O (CuO, Cu)	4.26	55	irregular	443 ± 234
Glu-3	sulfate	9.6 ^a)^	1.8	80 ^a)^	70	dropwise addition of a 12 M NaOH solution (20 mL; delivery time: 5 min). NaOH, Glu.	10	Cu_2_O (CuO)	4.27	73	truncated octahedral	2037 ± 295
Glu-4	sulfate	9.6	1.8	80	70	NaOH in pellets. NaOH, Glu.	10	Cu_2_O (Cu)	4.28	59	octahedral	3016 ± 444
Glu-5	sulfate	9.6	1.8	120	70	NaOH in pellets. NaOH, Glu.	10	Cu_2_O (CuO)	4.26	72	octahedral	2957 ± 375
Glu-6	sulfate	9.6	1.8	200	70	NaOH in pellets. NaOH, Glu.	10	Cu_2_O (CuO)	4.26	64	truncated octahedral	3179 ± 1516
Glu-7	sulfate	9.6	1.8	100	RT	NaOH in pellets. NaOH, Glu.	10	Cu_2_O (CuO)	4.26	67	octahedral	3375 ± 471
Glu-8	sulfate	9.6	1.8	100	50	NaOH in pellets. NaOH, Glu.	10	Cu_2_O (CuO)	4.27	75	truncated octahedral	2649 ± 797
Glu-9	sulfate	9.6	1.8	100	90	NaOH in pellets. NaOH, Glu.	10	Cu_2_O (CuO, Cu)	4.26	76	truncated octahedral	2103 ± 329
Glu-10	sulfate	9.6	0.9	100	70	NaOH in pellets. NaOH, Glu.	10	Cu_2_O (CuO)	4.29	47	truncated octahedral	3456 ± 469
Glu-11	sulfate	9.6	3.6	100	70	NaOH in pellets. NaOH, Glu.	10	Cu_2_O, Cu	4.26	53	truncated octahedral	1233 ± 434
Glu-12	sulfate	4.8	1.8	100	70	NaOH in pellets. NaOH, Glu.	10	Cu_2_O	4.26	71	truncated cube	1532 ± 548
Glu-13	sulfate	4.8	0.9	100	70	NaOH in pellets. NaOH, Glu.	10	Cu_2_O (CuO)	4.27	73	truncated cube	2065 ± 591
Glu-14	sulfate	4.8	3.6	100	70	NaOH in pellets. NaOH, Glu.	10	Cu_2_O (CuO)	4.25	64	truncated cube	779 ± 117
Glu-15	nitrate	9.6	1.8	100	70	NaOH in pellets. NaOH, Glu.	10	Cu_2_O (Cu)	4.26	71	octahedral	2194 ± 513
Glu-16	acetate	9.6	1.8	100	70	NaOH in pellets. NaOH, Glu.	10	Cu_2_O (Cu)	4.27	65	octahedral	3611 ± 1282
Glu-17	nitrate	4.8	0.9	100	70	NaOH in pellets. NaOH, Glu.	10	Cu_2_O, CuO	4.26	57	octahedral	721 ± 85
Glu-18	acetate	4.8	0.9	100	70	NaOH in pellets. NaOH, Glu.	10	Cu_2_O	4.27	68	polyhedral	4101 ± 1758

a In this synthesis (Glu-3), the amount of NaOH is delivered in the form of a 12 M aqueous solution (i.e., solubilizing 9.6 g of NaOH in 20 mL of deionized water) and added dropwise to the Cu­(II) aqueous solution (80 mL), thus maintaining the total volume of water of 100 mL.

#### 2.1.3 Reaction Temperature

The effect of the reaction temperature was studied by repeating the synthesis at different temperature conditions, namely: at room temperature (RT) (Glu-7), 50 °C (Glu-8), 70 °C (Glu-1), and 90 °C (Glu-9). Figure 4 reports the SEM micrographs of cuprite particles obtained by varying reaction temperatures, whereas the relative XRPD analysis is reported in the Supporting Information (Figure S5). From Figure 4, it appears that the particles’ morphology strongly depends on the synthesis temperature. In fact, the temperature represents an important thermodynamic and kinetic parameter in solution-mediated crystal growth because it simultaneously affects precursor dissolution, reduction kinetics, supersaturation, mass transport, nucleation, and facet-dependent growth rates. In the present system, increasing the temperature does not produce a monotonic evolution of the Cu_2_O habit. Octahedral crystals with straight well-defined edges were observed when the synthesis is performed at RT (Glu-7, ca. 3.37 ± 0.47 μm; Figure 4a). By progressively increasing the reaction temperature, {100} crystal planes enter the crystal morphology, thus resulting in the formation of (100)-truncated octahedra when the synthesis is performed at 50 °C (Glu-8, ca. 2.65 ± 0.80 μm; Figure 4b). As the temperature rises further, there is a tendency toward the formation of large octahedra with rounded edges at 70 °C (Glu-1, ca. 3.57 ± 0.43 μm; Figure 4c), whereas at 90 °C and slightly {100}-truncated octahedral morphology reappears (Glu-9, ca. 2.10 ± 0.33 μm; Figure 4d). Such behavior can be rationalized by considering the competition between thermodynamic surface stabilization and kinetically controlled growth. According to the literature, temperature is a fundamental thermodynamic parameter influencing crystal growth, both in terms of the growth rate and morphology, as each crystal form has its own kinetic law.^26^ In particular, by increasing the reaction temperature, the morphological importance of different crystal faces can vary significantly.^48^

**Figure 4:**
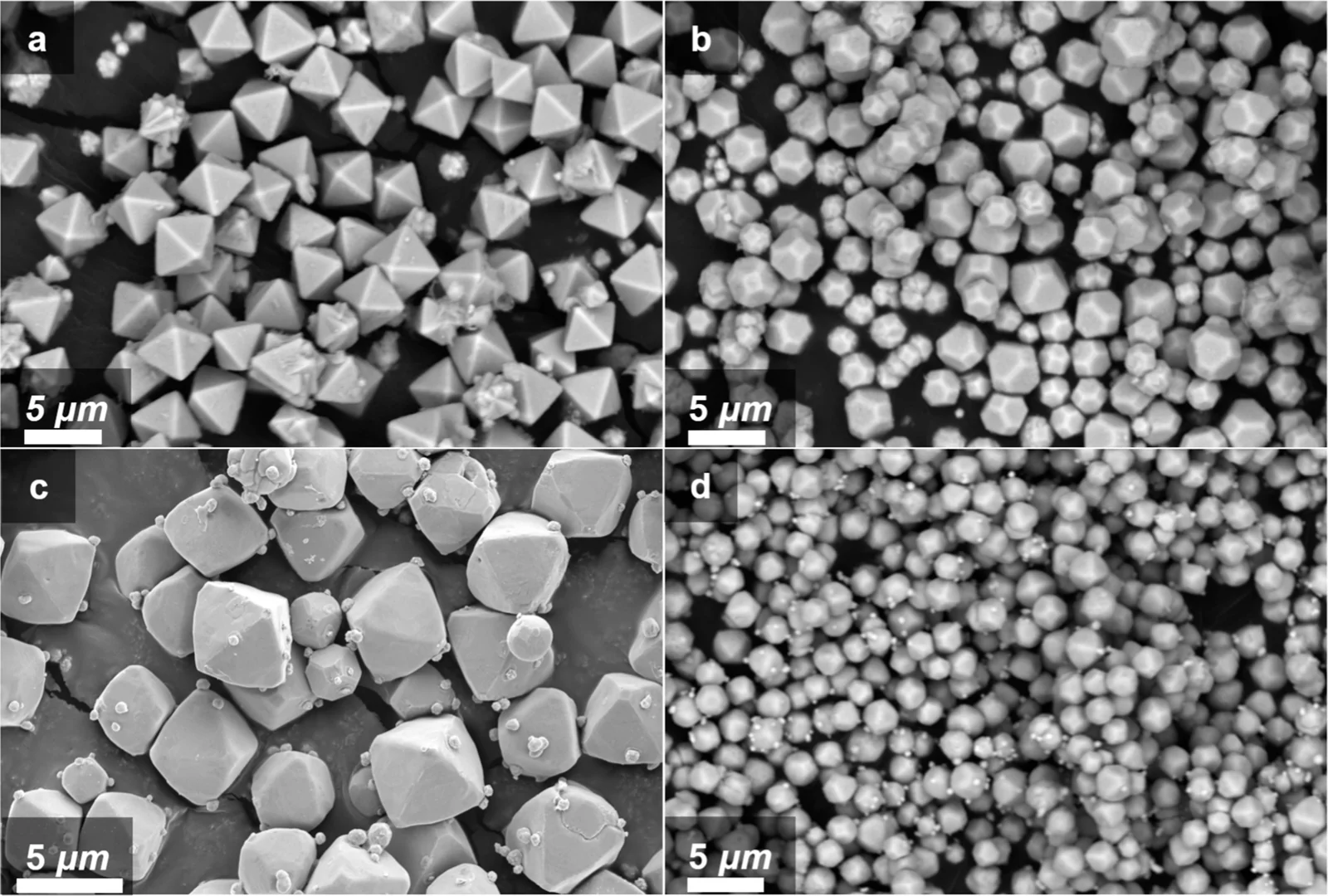
SEM micrographs of Glu-samples: effect of the reaction temperature, namely: (a) Glu-7 (RT), (b) Glu-8 (50 °C), (c) Glu-1 (70 °C), and (d) Glu-9 (90 °C). All micrographs were collected at the same magnification (10k ×).

An increment of the temperature enhances the diffusion and migration of Cu-containing species and growth units, thereby modifying the relative growth rates of the {111} and {100} facets. At the same time, the temperature-dependent reduction of Cu­(II) by glucose changes the supersaturation history and consequently the balance between nucleation and crystal growth. Therefore, the final morphology reflects the combined effect of these processes rather than a simple monotonic dependence on temperature. This interpretation is consistent with previous studies by Bai et al.^49^ showing that temperature can alter the relative development of {100} and {111} surfaces in Cu_2_O crystals. In particular, enhanced diffusion and surface migration at increasing temperature can favor faster growth along selected crystallographic directions, while at sufficiently high-temperature-enhanced surface rearrangement and dissolution/recrystallization may again modify the relative stability of the exposed facets. The recurrence of truncated octahedra at 90 °C in the present system may therefore result from a renewed change in the balance between {111} and {100} facet growth rather than from a simple continuation of the trend observed at an intermediate temperature. XRPD analysis confirmed the occurrence of Cu_2_O crystal phase for all samples, with traces of CuO and metallic Cu crystal phases (the latter one at high temperatures; Figure S5).

#### 2.1.4 Quantity of Reactants

To verify the effect due to the variation of quantity of reactants on morphology and average particle size, the amounts of precipitating agent NaOH and the capping/reducing agent glucose present in the solution were varied with two quantities of NaOH (either 4.8 or 9.6 g) and three quantities of glucose (either 0.9, 1.8, or 3.6 g). Figure 5 reports the SEM micrographs of such systems, whereas the relative XRPD analysis is reported in the Supporting Information (Figure S6). When employing larger amounts of NaOH (i.e., 9.6 g), glucose seems to be influencing the morphology, favoring a transition from octahedral particles with small {100}-truncations at low glucose content (Glu-10, ca. 3.46 ± 0.47 μm; Figure 5a), to large octahedral particles at intermediate glucose content (Glu-1, ca. 3.57 ± 0.43 μm; Figure 5b), toward octahedra with {100} and {110} truncations plus anhedral/irregular crystals with few individuals bearing {100}, {111} and {110} faces at high glucose content (Glu-11, ca. 1.23 ± 0.43 μm; Figure 5c). Interestingly, in the case of crystals obtained employing half the amount of NaOH (i.e., 4.8 g), it favored the transition toward a cube morphology with {111} and {110} truncations (Glu-13, ca. 2.06 ± 0.59 μm; Figure 5d), {100} dominating with {110} and definitely smaller {111} facets (Glu-12, ca. 1.53 ± 0.55 μm; Figure 5e), and, finally, cuboctahedra without rhombic dodecahedra faces (Glu-14, ca. 779 ± 117 nm; Figure 5f). This result demonstrates that the amount of NaOH is one of the main factors in determining both morphology and average particle size, through its effect on the chemical environment (pH) and supersaturation of the system. Hence, by halving the amount of NaOH, it was registered (i) a decrease in the average particle size, which is consistent with an increased nucleation frequency, and (ii) an increased occurrence of {110} surfaces alongside {100} and {111} facets. Increasing the NaOH concentration modifies the solution chemistry, consequently, the relative growth rates of the different Cu_2_O crystallographic facets. This effect may involve changes in copper speciation, supersaturation, glucose oxidation kinetics, and the formation and surface interaction of gluconate species. The emergence of {110} facets is therefore interpreted as a consequence of the modified growth kinetics of the {100}, {110}, and {111} facets rather than as a direct stabilization effect of hydroxide ions. These results agree with the study by Yang et al.^50^ that synthesized Cu_2_O crystals employing SDS as a structure directing agent and hydroxylamine hydrochloride as a reducing agent. In their study, they investigated the effect due to the hydroxide ion concentration in tailoring the Cu_2_O crystal morphology, registering a transition from rhombic dodecahedron to spherical, octahedral-like and then to hexapod particles upon the increase in the NaOH concentration. These results suggested that a lower pH favors the formation of rhombic dodecahedral crystals exposing {110} facets. Yang et al.^50^ attributed this behavior to the higher positive character of the {110} surfaces, associated with a larger number of Cu dangling bonds, which may favor adsorption of ionic species under conditions of lower hydroxide ion concentration.

**Figure 5:**
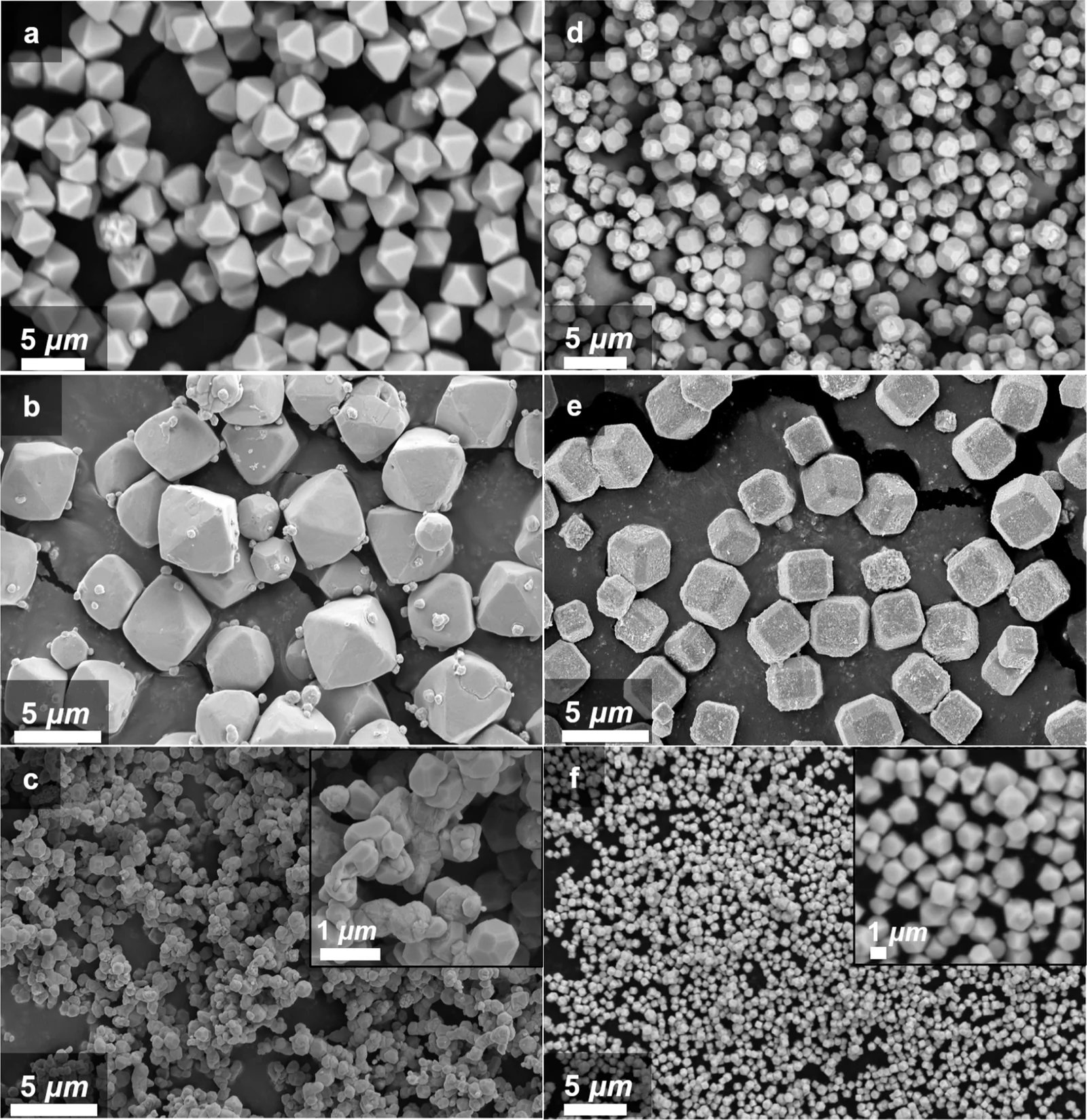
SEM micrographs of Glu-samples: effect of the quantity of reactants, namely: (a) Glu-10 (NaOH 9.6 g, Glu 0.9 g), (b) Glu-1 (NaOH 9.6 g, Glu 1.8 g), (c) Glu-11 (NaOH 9.6 g, Glu 3.6 g), (d) Glu-13 (NaOH 4.8 g, Glu 0.9 g), (e) Glu-12 (NaOH 4.8, Glu 1.8 g), and (f) Glu-14 (NaOH 4.8, Glu 3.6 g). All micrographs were collected at the same magnification (10k ×), whereas the insets in panels (c,f) were collected at high magnification (30k ×).

Considering the capping/reducing agent glucose, increasing its amount from 0.9 to 1.8 g, independently from the NaOH concentration, produced an almost constant particle size, followed by a sharp size reduction with 3.6 g of glucose. Overall, the presence of glucose (together with its oxidation product, gluconate anion) appears to enhance the morphological importance of the {100} surfaces, an effect superimposed on the strong influence of NaOH and more evident at low hydroxide concentrations. The greatest importance for {100} is mainly observed at low NaOH levels and intermediate concentrations of glucose (Glu-12; Figure 5e). XRPD analysis confirmed the occurrence of Cu_2_O crystal phase for all samples, with relevant metallic Cu content at high reducing agent glucose content (see, for instance, the XRPD pattern of the Glu-11 sample in Figure S6c).

Regarding the role of gluconate, Huang et al.^51^ investigated the synthesis of Cu_2_O crystals using Cu­(II) sulfate as a copper precursor, glucose as a reducing/capping agent, and NaOH as a precipitating agent. By varying the NaOH concentration, the authors observed a systematic evolution of the crystal morphology from cubes, exposing {100} facets, to cuboctahedra, truncated octahedra, and finally octahedra dominated by {111} facets. According to their interpretation, the NaOH concentration affects the relative growth rates of the different crystallographic facets, while the oxidation of glucose during the reduction process consumes hydroxide ions and produces gluconate species. The authors further observed an increase in the average crystal size with increasing glucose concentration, which was attributed to the influence of glucose on crystal growth. In this context, the formation of gluconate was proposed to contribute to the modification of the growth environment and, consequently, to the evolution of crystal morphology. These findings are relevant to the present study, although the morphological evolution observed here does not completely reproduce that reported by Huang et al.^51^ In our experiments, increasing the glucose concentration resulted in a decrease in particle size, together with an increased contribution of {100} surfaces, particularly at low NaOH concentrations and intermediate glucose loadings. This behavior can reasonably be related to the consumption of hydroxide ions associated with glucose oxidation and the consequent formation of gluconate species. However, glucose, gluconate, and NaOH are intrinsically interconnected in the present reaction system, making it difficult to isolate the individual contributions of each component. Therefore, while the literature supports a possible role of glucose oxidation products in modifying Cu_2_O crystal growth, the present experimental evidence does not allow us to establish a direct relationship between gluconate adsorption and stabilization of a specific crystallographic facet.

#### 2.1.5 Cu Precursors

Lastly, the effect of the different Cu^2+^ precursors was monitored by replacing the sulfate precursor with either nitrate or acetate salts, maintaining constant the molar concentration of Cu^2+^ ions. Two syntheses were considered, namely: Glu-1 (curved octahedral particles) and Glu-13 (truncated cubes). SEM micrographs of such systems, obtained by varying the nature of the Cu^2+^ precursor, are reported in Figure 6, whereas the relative XRPD analysis is reported in the Supporting Information (Figure S7). Concerning the Glu-1 synthesis, the replacement of sulfate salt with other precursors does not seem to have particularly affected the particle morphology, which remains octahedral in both cases (Figure 6a–c). The main effect is the change in curvature of the edges, which is absent for the synthesis with the acetate precursor (Glu-16 sample, ca. 3.61 ± 1.28 μm; Figure 6c). Interestingly, sample Glu-15 (Figure 6b) closely resembles the already discussed sample Glu-5 (Figure 2c), showing the presence of high-index forms {32*l*} (more likely with *l* = 1). Moreover, the use of nitrate precursor remarkably affects the average particle size, causing a reduction from ca. 3.57 ± 0.43 μm (Glu-1; sulfate precursor) to ca. 2.19 ± 0.51 μm (Glu-15; nitrate precursor).

**Figure 6:**
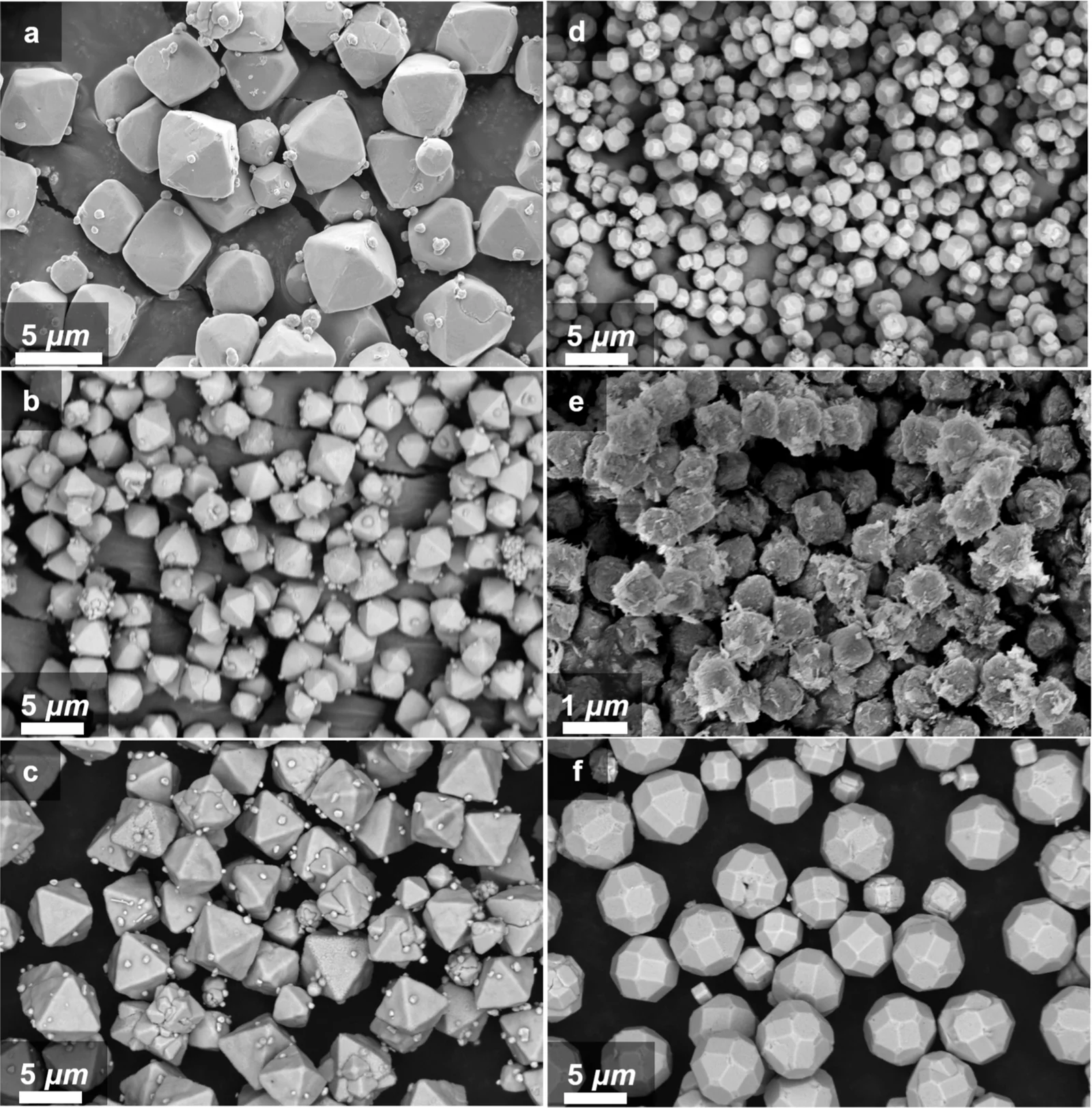
SEM micrographs of Glu-samples: effect of the Cu precursors, namely: (a) Glu-1 (sulfate, NaOH 9.6 g, Glu 1.8 g), (b) Glu-15 (nitrate, NaOH 9.6 g, Glu 1.8 g), (c) Glu-16 (acetate, NaOH 9.6 g, Glu 1.8 g), (d) Glu-13 (sulfate, NaOH 4.8 g, Glu 0.9 g), (e) Glu-17 (nitrate, NaOH 4.8, Glu 0.9 g), and (f) Glu-18 (acetate, NaOH 4.8, Glu 0.9 g). All micrographs were collected at the same magnification (10k ×), with the only exception of panel (e), which was collected at high magnification (30k ×).

Vice versa, in the case of Glu-13 synthesis, the replacement of sulfate salt with other precursors significantly affects not only the average particle size but also their morphology. In particular, if the use of the sulfate precursor leads to the formation of {111} and {110} truncated cubes of ca. 2.06 ± 0.59 μm (Glu-13; Figure 6d), the nitrate precursor allows obtaining octahedral particles of ca. 721 ± 85 nm with dirty surfaces (Glu-17; Figure 6e) probably contaminated by lamellar tenorite crystals, whereas the acetate precursor leads to the formation of regular polyhedral particles of ca. 4.10 ± 1.76 μm (Glu-18; Figure 6f) with the copresence of {110}, {111}, and {100} surfaces of similar morphological relevance. However, crystals of sample Glu-18 also exhibit tiny {211} facets (Figure S3), similarly to sample Glu-6 (Figure 3d). In Glu-18, the {211} surfaces are less relevant, with enlarged {110} faces and reduced {111}. Structural analysis revealed in all cases, the presence of Cu_2_O crystal phase with signals due to CuO in the case of the Glu-17 sample (nitrate precursor; Figure S7). The occurrence of CuO is likely due to the oxidizing properties of nitrate ions.

### 2.2 Morphology Influence on Cuprite Induced by Employing PVP/Ascorbic Acid

The following paragraph is focused on the investigation of the effects of compositional and experimental parameters on the final morphology of Cu oxides produced by using PVP as a capping agent, AA as a reducing agent, and NaOH as a precipitating agent. With respect to the previous synthesis, which used glucose as a single capping/reducing agent, both capping and reducing agents were added separately in the following protocol. Additionally, the Cu^2+^ precursor selected for this chemical route is the acetate salt, whereas in the previous synthesis, the sulfate salt was used. The reason behind this choice is starting from the protocol of synthesis of Bai et al.^49^ found in the literature.

#### 2.2.1 Initial Volume of Deionized Water

As previously discussed, the effect due to pH variation induced by concentration was monitored by varying the volume of reaction (i.e., maintaining constant molar ratios of all the reagents). Figure 7 reports the SEM micrographs of cuprite particles obtained by varying the initial volume of deionized water, whereas the relative XRPD analysis is reported in the Supporting Information (Figure S8). In particular, the water volume was varied as follows, namely: 500 mL (PA-4; Figure 7a), 400 mL (PA-1; Figure 7b), 200 mL (PA-3; Figure 7c), and 100 mL (PA-2; Figure 7d). XRPD analysis confirmed in all cases the occurrence of the Cu_2_O crystal phase, with the presence of a large amorphous signal centered at ca. 25°2θ in the case of the quasi-spherical PA-2 sample (Figure S8d).

**Figure 7:**
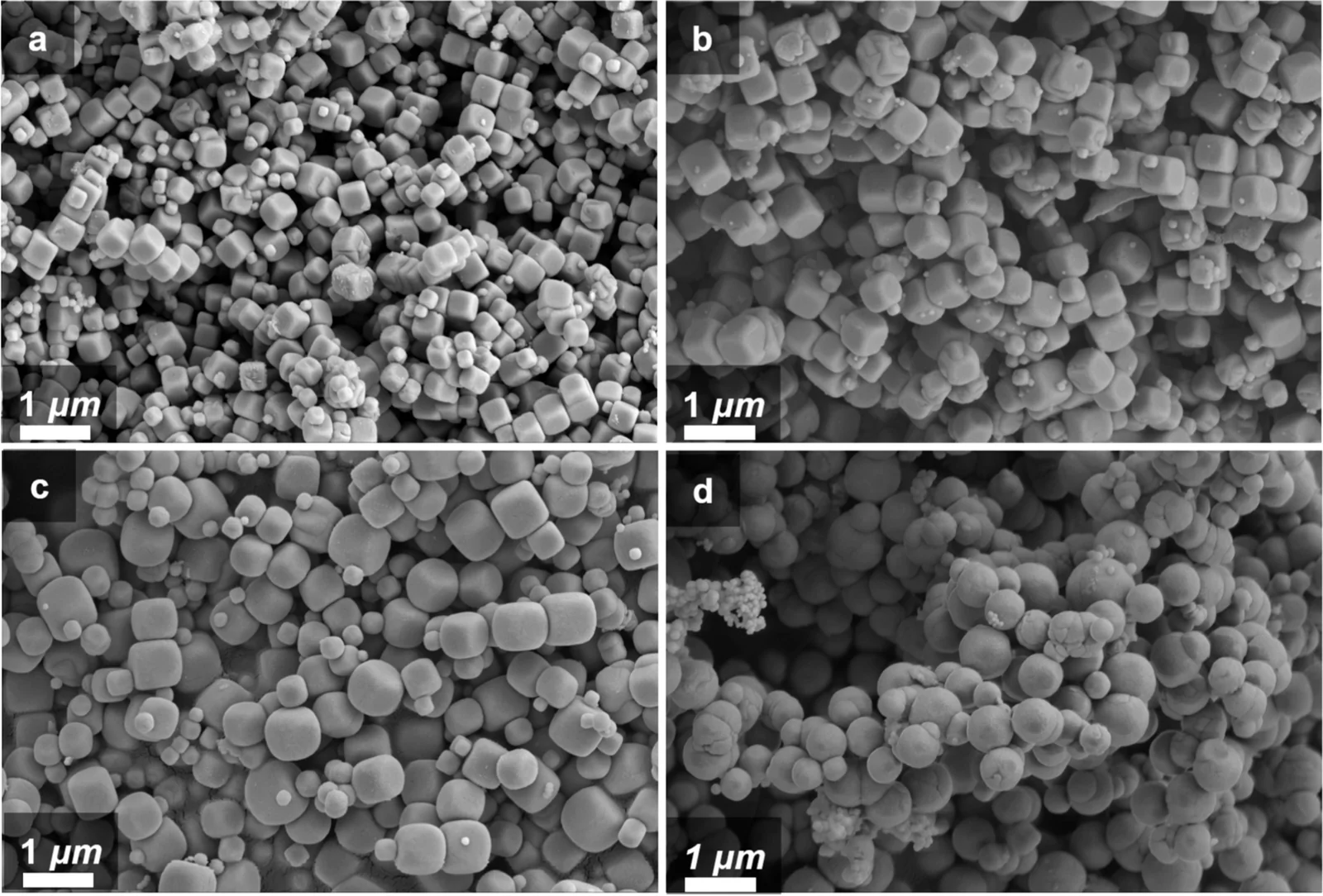
SEM micrographs of PA samples: effect of the initial volume of deionized water, namely: (a) PA-4 (500 mL), (b) PA-1 (400 mL), (c) PA-3 (200 mL), and (d) PA-2 (100 mL). All micrographs were collected at the same magnification (30k ×).

The morphological analysis revealed a gradual transition from small cubes (PA-4 and PA-1, ca. 283 ± 117 nm and ca. 349 ± 165 nm, respectively; Figure 7a,b) to intermediate cubes with rounded edges and surfaces (PA-3, ca. 447 ± 241 nm; Figure 7c) to large spherical particles (PA-2, ca. 550 ± 237 nm; Figure 7d) by decreasing the dilution volume, thus confirming the effect on both the morphology and the average particle size. However, with respect to the previous protocol, where the dilution favored the formation of more rounded surfaces, in the present system the dilution effect favored the formation of cube systems with better defined edges and flatter surfaces. This result can be related to the variation of the reaction volume, while keeping the moles of reagents constant, which alters concentrations and chemical interactions between the different species present in the solution, including adsorption phenomena on the surface of the particles. Furthermore, by decreasing the water volume within the solution, a variation in the solution pH was registered, going from ca. 12.9 for the PA-4 sample to ca. 13.6 for the PA-2 sample. In this context, as previously reported, pH is a key factor for controlling both nucleation and growth rate of particles due to their ability to complex the species present in solution.^52^

The crystal morphologies observed in Figure 7 deserve a detailed analysis due to the presence of heavily rounded edges and surfaces, features that are normally in contrast with the anisotropic nature of crystal structures and crystal growth mechanisms. However, it is well known that additives/impurities strongly interacting with the growing surfaces can trigger dramatic morphological changes,^19,26,53^ with biominerals being the most amazing example of this outcome.^54^ Starting from sample PA-4, synthesized in the presence of 7.5 mmol L^–1^ of PVP-10k (expressed as monomeric units, ca. 90 monomers per chain), a significant level of rounding of the crystals is observed, suggesting an active role of the PVP molecules in the solution. The Cambridge Structural Database stores a few structures of copper compounds with four bridging carboxylato groups and pyrrolidin-2-one ligands,^55–58^ where the dimer motif of Cu­(II) acetate monohydrate is maintained. Copper acetate is characterized by pentacoordinated Cu­(II) ions with four bridging acetate groups and the fifth coordination site filled by a water molecule. Therefore, the presence of PVP-10k polymeric molecules can be highly effective at solvating copper species in solutions containing acetate and gluconate anions and, more relevant for the present study, also capable of strong interactions with cuprite crystal surfaces. The interactions of PVP chains with copper surface sites on growing crystals can specifically alter the growth rates of different surfaces, leading to poisoning or blocking of kink sites, ledges, or terraces.^19^ This effect, in turn, reduces the advancement rate of surfaces, making them more relevant in the final growth morphology. Thus, high-index faces, that are normally absent from the growth morphology due to their fast growth, can appear and even become morphologically dominant.

The TEM images of Figure 8 for sample PA-3 clearly show profiles of the cubic crystal habit, complementing information gathered by SEM analysis (Figure 7). The rounded edges connecting faces of the {100} form are evident. On increasing the magnification (Figure 8a–e), the profiles of the crystals show more detail. Edges that appear curved at low resolution start to break down into shorter straight edges, suggesting a dense collection of high-index faces all belonging to the same ⟨100⟩ zone axis. Thus, the 12 sharp edges of a cube are here better described as a group of {*h*10} faces with *h* ≥ 1, *h* = 1 being the rhombic dodecahedron. Analysis of TEM projections of TEM images allowed the extraction of the Miller indices of the microcrystals of cuprite.^59,60^

**Figure 8:**
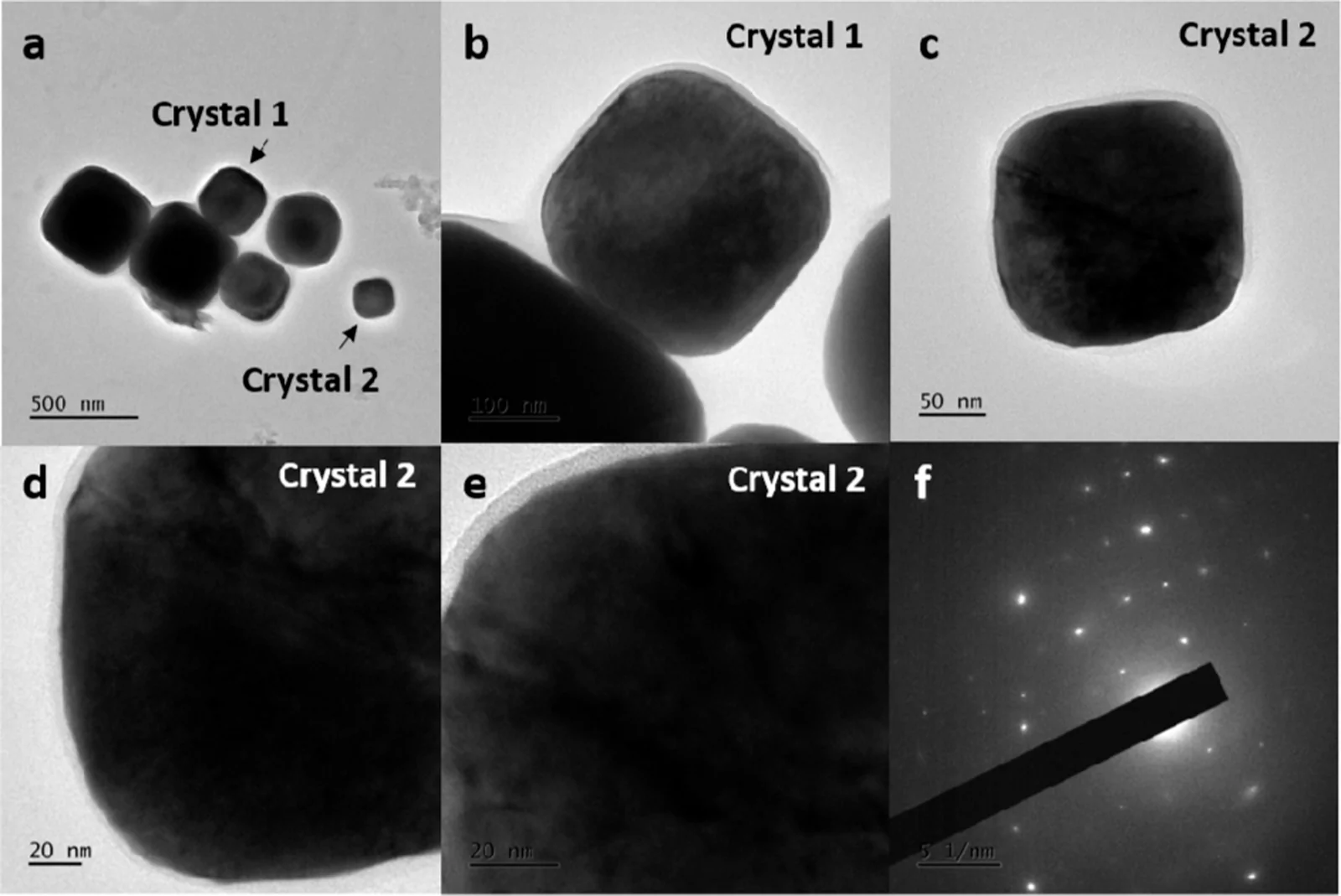
TEM images of the PA-3 sample: (a) survey at low magnification, (b) image of crystal 1 at medium magnification, (c–e) images of crystal 2 at medium–high magnifications, and (f) SAED analysis.

The same phenomenon is responsible for the smoothing of the eight vertices of a cube (evident in Figure 7), where the presence of PVP macromolecules makes a group of {*h*11} faces with *h* ≥ 1 appear in the growth morphology. The crystal model of Figure 9 shows these features within the limits of *h* ≤ 4 and *h* ≤ 3 for {*h*10} and {*h*11} faces, respectively.

**Figure 9:**
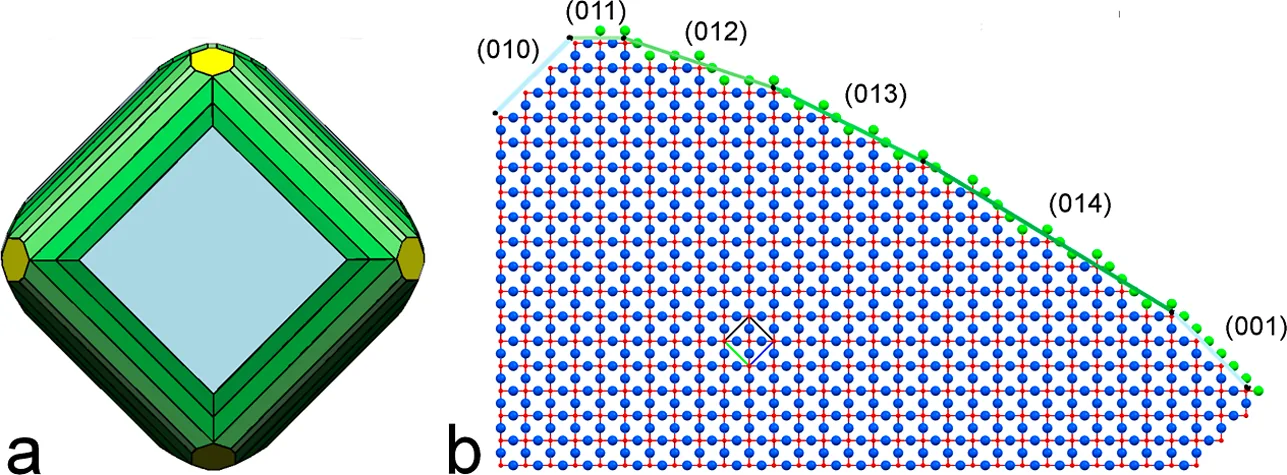
(a) Model of cuprite crystal for sample PA-3 viewed down [100], with dominant {100}, small {111}, and {*h*10} forms with *h* ≤ 4. Color coding of forms can be found in Figure S9. (b) Projection of cuprite crystal structure down [100], showing half of the [100] edge built from several {01*l*} forms. Dihedral angles between adjacent faces: (011) ∠ (012) 18.4°; (012) ∠ (013) 8.1°; (013) ∠ (014) 4.4°; (014) ∠ (001) 14.0°. Color coding for atom species: red = O, blue = fully coordinated Cu, light green = dangling Cu. Crystallographic cell axes: green *b*, blue *c*.

Real cuprite crystals, such as those of Figure 7, likely involve surfaces with even higher indexes. We suggest that PVP acts as an effective growth additive on several crystallographic planes. This performance is correlated to the polymeric nature of the additive, comprising either ca. 90 monomeric units for PVP-10k or ca. 500 monomeric units for PVP-55k, linked in a conformationally flexible chain with C=O Lewis basic sites.

However, we also estimate the fundamental role of the crystal structure of the cuprite. The structure of cuprite owns the high cubic symmetry of space group P*n*3̅*m* (no. 224) and comprises two interpenetrated networks without direct bonding between them. The Cu­(I) centers have a diagonal coordination with two oxide ions, while the oxygen atoms interact with four Cu­(I) with a tetrahedral stereochemistry. This indicates that the cuprite crystal structure is compact with short Cu–O bond distances of ca. 1.84 Å and Cu···Cu first neighbors are at 3.01 Å, in a small unit cell with *a* = 4.259 Å.^61^ Little is known about the surface structure of cuprite crystals outside of a high vacuum. Modeling of Cu_2_O {100} and {111} surfaces interacting with water molecules shows complex behavior with preference for dissociative adsorption on top of oxide anions or Cu^+^ centers for {100}^62^ and molecular chemisorption of H_2_O favored over the dissociative one for {111}.^63^ Cuprite is known for having at least 20 different crystal forms observed in natural crystals,^46^ exhibiting a wide variety of habits and morphologies (reproduced in Figure S4).^47^ Therefore, it is not surprising to find extreme flexibility in producing complex morphologies based on high-index planes in synthetic crystals. Recently, convex polyhedra with high index faces have been synthesized. These polyhedra include, beside differently developed {100}, {110}, and {111} faces, well evident high index {211}, {311}, {331}, {522}, and {544} facets.^59,60,64,65^

In Figure 9b, the crystal structure of cuprite is projected along the [100] direction. Different crystallographic planes having [100] as a zone axis are also represented and highlighted (see colored lines in Figure 9b). The cube faces (010) and (001) enclose this sequence of planes. In between these boundaries, a group of (01*l*) surfaces are cut from the bulk structure with dangling Cu­(I) ions (light green colored atoms in Figure 9b). From the figure, it can be appreciated that the structure of the surfaces becomes more complex and rougher as the Miller *l* index increases, independently from considering or neglecting the dangling Cu atoms (possibly stabilized by water molecules, OH^–^ ions or PVP macromolecules).^62,63^ Neglecting the Cu­(I) dangling atoms reveals that different lengths of steps along the horizontal ([011]) and vertical ([011̅]) directions provide the right orientation for the (01*l*) surfaces. Such surface structural models have been long accepted and exploited with many examples nicely fitting this point of view.^66^ Among important crystalline substances, tetragonal crystals of ammonium or potassium dihydrogen phosphate have a growth morphology comprising a {100} prism and {011} pyramid that can be heavily altered by the presence of trivalent metal cations. The wedge-like-shaped (tapered) crystals with new (10*l*) orientations arise from retardation of the growth of {100} faces enhanced by impurities.^19^ The present authors consider that PVP is able to retard many different growth surfaces (at least {*h*10} and {*h*11}) that would otherwise be absent from the final growth morphology due to their high growth rate. However, in the absence of any experimental data related to the adsorption isotherm of PVP on cuprite crystals, it is only possible to estimate the number of available pyrrolidone monomeric units per surface Cu atoms. Assuming spherical crystals with an average size of ca. 500 nm and considering a surface density of Cu atoms of 1.27 × 10^15^ cm^–2^ (calculated for the {111} surfaces, which show the highest density of surface Cu atoms), with 4.5 mmol of PVP monomers (i.e., 2.71 × 10^21^ monomers), there are ca. 600 pyrrolidone C = O binding sites (likely not all available at the same time due to steric constraints) per surface Cu atom available in solution. Such a ratio makes it reasonable to assume a great potential activity of PVP macromolecules during the growth of cuprite crystals.

In this context, authors recall here that crystal growth morphology ensues from the interplay of crystallographic anisotropy and growth mechanisms and kinetics by means of interfacial processes (together with long-range transport phenomena not here considered).^67^ Growth under the control of interfacial kinetic processes provides a “kinetic Wulff shape” based on the attachment energy released upon capture of the growth units.^17,68^ The equilibrium crystal morphology, on the contrary, minimizes the (anisotropic) surface free energy of the crystal under the constraint of constant volume and represents an equilibrium property of the system and differs from the growth morphology.

Therefore, the growth process can be altered significantly even for very simple morphologies by changing the rate of advancement of faces. Natural cuprite samples are found with {111}, {110}, and {100} as the main forms plus {211}, {221}, {210}, {322}, and {510}, in order of decreasing probability.^69^

According to the structural classification of growing surfaces,^68^ the growth mechanisms of these surfaces depend on their flat (F faces), stepped (S faces), or kinked (K faces) nature as well as the value of the attachment energy.^17^ F faces have an average low attachment energy, hence low growth rate and high morphological importance. Hartman and Perdok analyzed the cuprite structure with periodic bond chain theory.^69^ This semiquantitative analysis attributed a flat character only to forms {111}, {110}, and {100}, with attachment energies increasing in the same order, in agreement with the decreasing morphological importance of these forms. The other five observed forms have instead a stepped character, which implies, under normal conditions, a higher growth rate and small morphological importance or absence from the growth morphology. As a matter of fact, many mineral species exhibit complex morphologies comprising high-index forms that should not appear because of their S or K character. The widespread presence of impurities or interfering species in the growth environment can provide clues to the deep alteration of the expected crystal morphology. Therefore, findings about the large number of faces and/or the presence of high-index faces are not surprising and unexpected, and this is in agreement with the literature.^70^ The polyhedron in Figure 9a, a simplistic model of real samples, comprises 146 facets. This result is consistent with the previously discussed ideas. In general, the use of effective capping agents such as PVP allows access to very complex polyhedra.

On increasing the concentration of PVP simply by reducing the reaction volume (i.e., 9 mmol L^–1^ for PA-1 sample and 15 mmol L^–1^ for PA-3 sample), the level of edges and vertices smoothing increases, as is evident in Figure 7, i.e., the capping activity increases with the concentration of PVP macromolecules. In this context, examples of microcrystals with smoothed edges and vertices can be found in different fields. Smoothed crystals have been observed for cuprite after treating Cu_2_O at 550 °C for 6 days in the presence of Cu metal and water vapor, an environment without known impurities (as in Palmer).^71^

The feature of the PA-4, PA-1, and PA-3 samples is the reduced nucleation rate on increasing the PVP concentration, as judged from the larger size of the crystalline particles in the same order. This effect can be attributed to the PVP macromolecules being able to interact with the first cuprite embryos that subsequently evolve to nuclei and mature crystals.^72^ In fact, according to the literature, PVP is considered to adsorb onto {111} facets more strongly than on {100} due to dangling Cu^+^ ions on {111} surfaces, thus suppressing to a larger extent the growth of the {111} planes compared to {100}.^73^

Finally, regarding the PA-2 sample (obtained with PVP 22.5 mmol L^–1^), the capping action affords unusual crystal morphologies, with the formation of quasi-spherical cuprite particles (Figure 7d, and Figure 10). At first sight, these particles could be considered spherulites, born in a high supersaturation environment because of the high concentration of reactants, producing high nucleation rates and morphological instabilities.^26^ However, the surface of the PA-2 particles is smooth differently from what is usually observed for spherulites. Few particles are intergrown, but this also happens in the other trials. TEM imaging (Figure 10) provides more details of the surface profiles. Figure 10a,b suggests that the particles are not actually spherical because short straight projections bound all particles. Upon increasing the magnification (Figure 10c), this hypothesis is confirmed. In Figure 10d,e, the resolution of crystal planes is obtained. Figure 10e provides an interplanar distance of ca. 2.46 Å corresponding to the {111} planes of the cuprite phase. In Figure 10f, SAED data are shown with diffraction spots comprising slightly misaligned contributions. Although the diffraction data do not support randomly oriented polycrystalline aggregates, the presence of mosaicity or low-angle crystallographic subdomains cannot be completely excluded on the basis of the present characterization.

**Figure 10:**
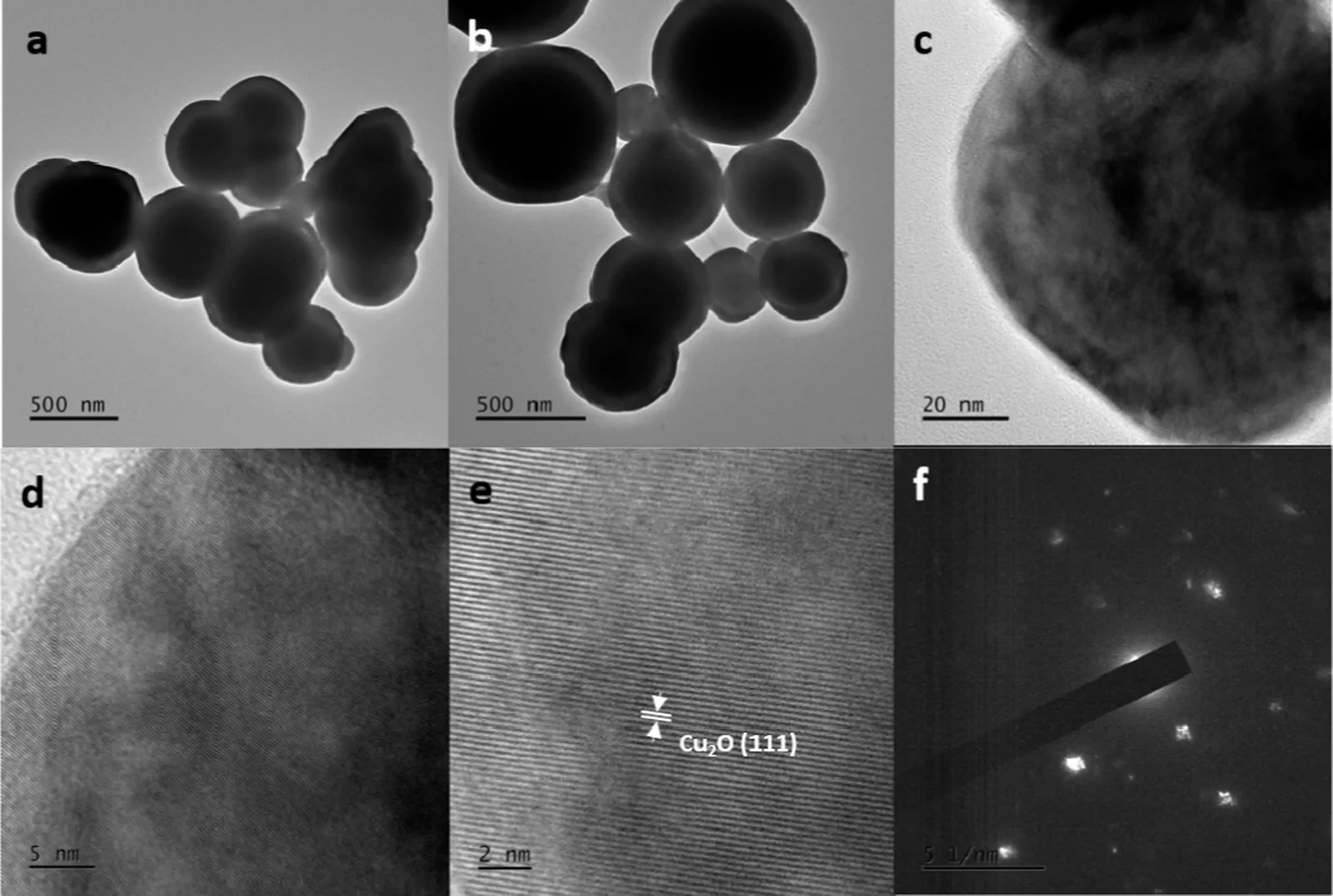
TEM images of the PA-2 sample: (a,b) survey at low magnification, (c) image at medium magnification, and (d,e) images at high magnifications with fringe pattern corresponding to the (111) plane of Cu_2_O (*d* = 2.46 Å; card number 01-078-2076), and (f) SAED analysis.

As reported in Figure S8d, the XRPD peaks are broadened and partially split into two components, indicating a contribution from amorphous material. At high concentrations of additives/impurities, it is often observed a negative effect on the FWHM of diffraction peaks due to an increased mosaic spread and reduction of the coherence length by the foreign substance entering the crystal structure.^54^ A similar effect could also be present in sample PA-1 (see Figure S8b), also showing peak broadening but without amorphization.

Hence, given the smooth trend of morphologies while changing the PVP concentration and other experimental evidence, the present authors consider sample PA-2 as crystalline, with partial degradation of the structural quality and representing the upper limit for the morphological change of cuprite crystals induced by PVP macromolecules. Analogously to what was previously discussed for the cubic particles with smoothed edges and vertices, the authors consider the PA-2 particles to be bounded by a series of high-index forms arising from the capping action of PVP macromolecules at high concentrations, the same mechanism as for the other samples but with a higher intensity. In fact, in the PA-2 case, new {*hkl*} forms, with *h*, *k*, and *l* all nonzero, can explain the quasi-spherical morphology. Each of these forms affords 48 symmetry-equivalent faces, producing polyhedral particles with extremely complex morphologies. Another system that consistently shows heavily anomalous morphologies is Barite (i.e., barium sulfate) in the presence of phosphonates. Under proper conditions, elliptical and disk-like crystals can be obtained due to specific interactions of organic phosphonates with crystal terraces.^74,75^

In Figure 11, the trend observed for increasing concentrations of reactants and PVP is summarized in a morphodrome picturing the crystal morphologies as a function of PVP concentration. The modeled morphologies represent a good approximation while involving a limited number of crystal forms. The actual situation is likely more complex, as SEM images show smooth surfaces without boundaries among the flat surfaces. The peculiar structural features of cuprite detailed above provide a sound explanation, where termination of surfaces can involve dangling Cu ions stabilized by solvent molecules, carboxylates (*e.g.,* acetate and gluconate), and pyrrolidone carbonyl groups or comprise small terraces of low index planes also stabilized by Lewis groups (Figure 9b).

**Figure 11:**
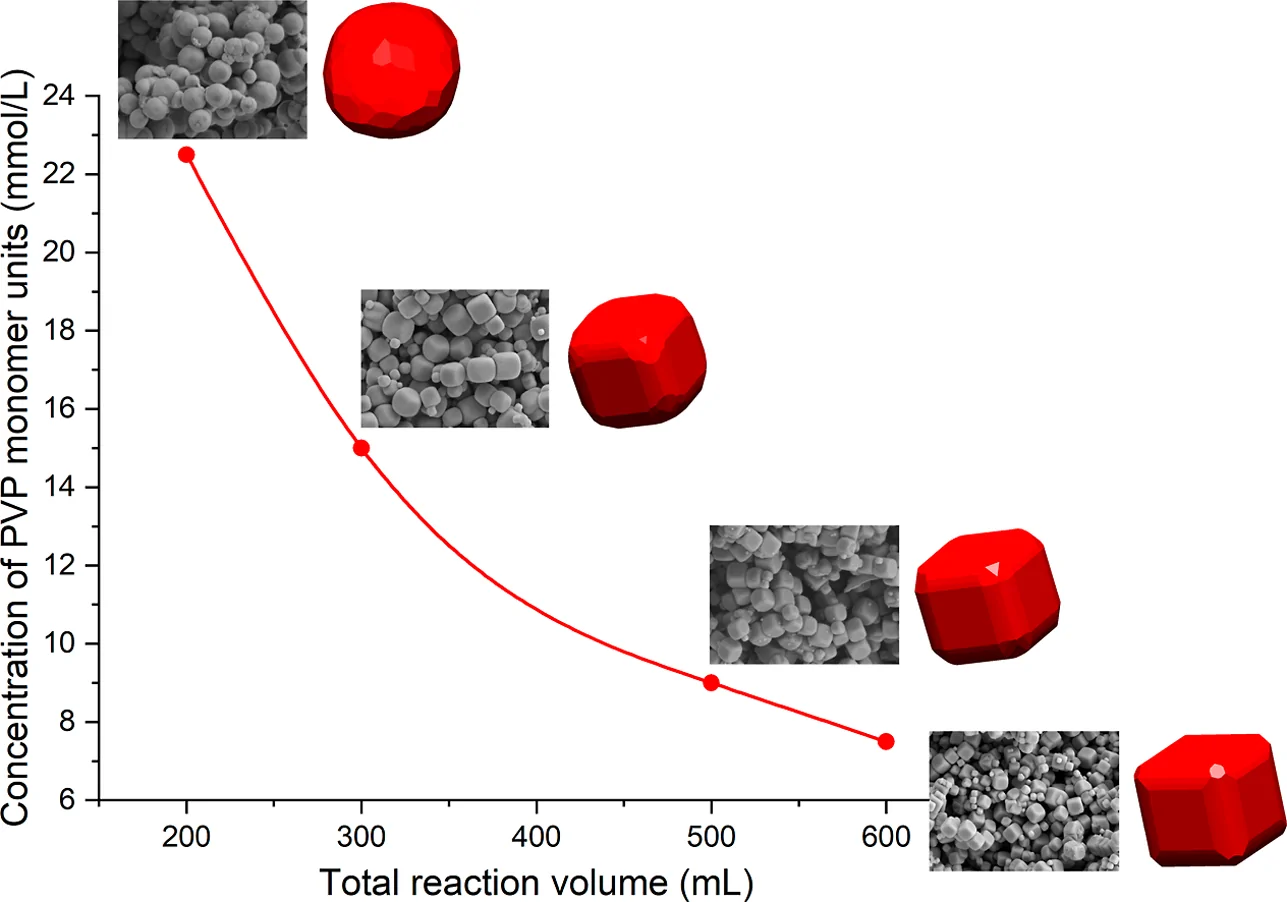
Morphodrome picturing the Cu_2_O crystal morphologies as a function of PVP concentration and the total reaction volume. The multifaceted crystals summarized in the morphodrome are also reported, with a color-coding of forms, in Figure S9, Supporting Information.

#### 2.2.2 Reaction Temperature

The effect of the reaction temperature was studied by repeating the synthesis at different temperature conditions, namely: at RT (PA-1), 50 °C (PA-5), and 75 °C (PA-6). Figure 12 reports the SEM micrographs of particles obtained by varying reaction temperatures, whereas the relative XRPD analysis is reported in the Supporting Information (Figure S10). In this particular system, independently from the reaction temperature, the cubic morphology was retained (Figure 12). Interestingly, the increment of temperature seems to be affecting (i) the average particle size, registering a decrease of ca. 50 nm, from PA-1 (at RT, ca. 349 ± 165 nm) to PA-6 (at 75 °C, ca. 290 ± 107 nm), and (ii) the surface of the cubes, registering the occurrence of less smooth surfaces as the synthesis temperature increases. XRPD analysis confirmed the occurrence of the Cu_2_O crystal phase for all samples, with traces of CuO at intermediate temperature, but probably due to the natural aging of the sample (Figure S10). Interestingly, Mishra et al.^76^ reported a synthesis protocol of Cu_2_O crystals employing Cu­(II) nitrate salt as a precursor in a DMF solvent, PVP as a capping agent, and sodium borohydride as a reducing agent. In their study, the authors maintained constant Cu­(II) nitrate concentration (i.e., 25 mM), and varied the reaction temperature from 40 to 140 °C, obtaining octahedra at 40 °C, truncated octahedra at 60 °C, truncated cubes at 80 °C, regular cubes at 100–120 °C, and truncated cubes with concave {100} facets at 140 °C. This suggests a change in the growth rate along different planes by varying the temperature condition. Conversely, in the PVP/AA-mediated protocol here investigated, no variation of the particles’ morphology was registered with temperature (i.e., with regular cube morphology within the entire temperature range investigated, from RT to 75 °C).

**Figure 12:**
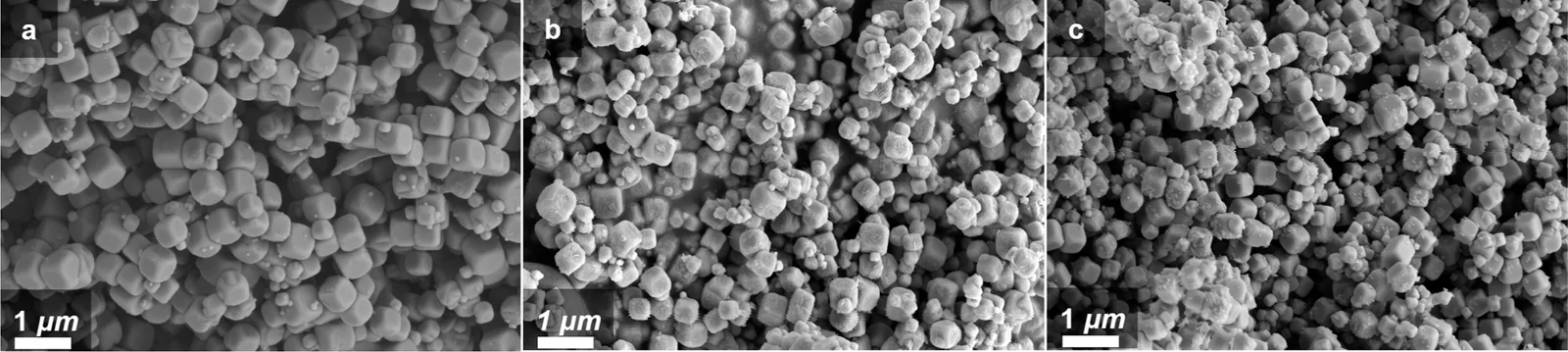
SEM micrographs of PA-samples: effect of the reaction temperature, namely: (a) PA-1 (RT), (b) PA-5 (50 °C), and (c) PA-6 (75 °C). All micrographs were collected at the same magnification (30k ×).

#### 2.2.3 Cu Precursors and PVP Molecular Weight

With the aim of investigating the potential effect due to the choice of precursors, a synthesis was carried out by changing the Cu^2+^ precursor by replacing the acetate salt with the nitrate salt while keeping constant the concentration of Cu^2+^ ions in solution. SEM analysis revealed that by changing the Cu^2+^ precursor from acetate (PA-1; Figure 13b) to nitrate (PA-7; Figure 13a), the general cubic morphology was retained, even if in the case of the PA-7 sample, together with cubic structures, the copresence of some elongated particles was registered. Regarding the average particle size, the use of the nitrate precursor caused an increment of the particles’ diameters by almost 100 nm (from ca. 349 ± 165 nm to ca. 446 ± 177 nm), opposite to what was registered in the glucose-mediated protocol. As previously discussed, the choice of precursors is a key parameter, as both the chemical species present in the solution and the complexes they form strongly influence nucleation and growth of crystals.^77^ In particular, the morphology of well-structured materials and their growth rate largely depend on the species adsorbed on the surface during their growth.^20^ Analogously to what was already registered in the glucose-mediated protocol, the structural analysis performed on the PA-7 sample (nitrate precursor; Figure S11) revealed the presence of the Cu_2_O crystal phase as the main principal phase with the copresence of CuO crystal phase as the secondary phase. This phenomenon could be explained considering the high oxidizing ability of nitrate ions with respect to their acetate counterpart.

**Figure 13:**
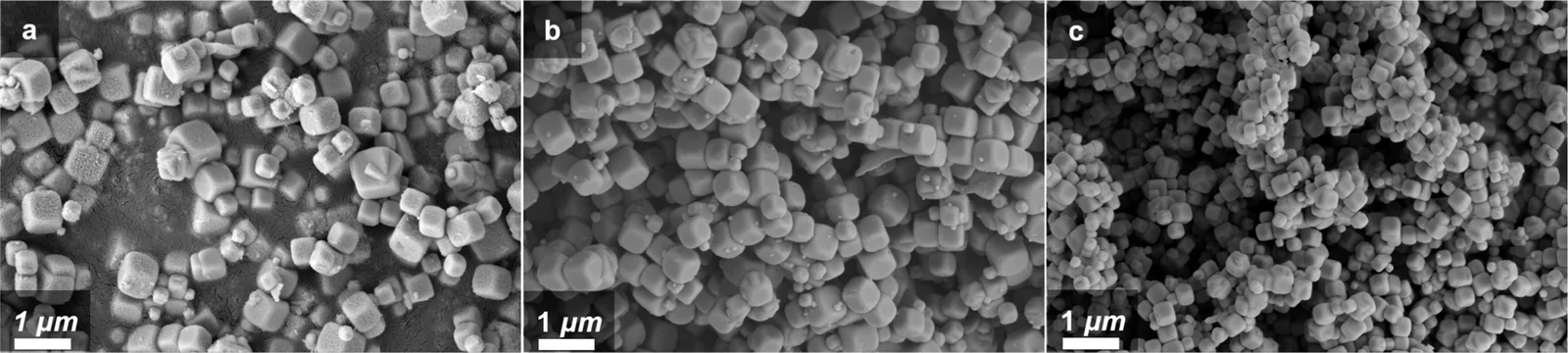
SEM micrographs of PA samples: effect of the Cu­(II) salts and PVP molecular weight, namely: (a) PA-7 (nitrate, PVP-10k), (b) PA-1 (acetate, PVP-10k), and (c) PA-8 (acetate, PVP-55k). All micrographs were collected at the same magnification (30k ×).

Interestingly, another aspect investigated is the chain length of the capping agent PVP. In fact, as previously reported, PVP is a water-soluble polymeric compound constituted by N-vinylpyrrolidone units and depending on the number of units forming the macromolecular chain, it can be obtained under different molecular weights.^78^ In the present protocol, two different molecular weights were investigated while maintaining constant the amount of polymer added, namely: PVP-10k (PA-1, ca. 349 ± 165 nm; Figure 13b) and PVP-55k (PA-8, ca. 259 ± 78 nm; Figure 13c). SEM micrographs pointed out that the cubic morphology has been retained, whereas a significant change in the average particle size was recorded, registering a decrease of ca. 100 nm employing the PVP with higher molecular weight.

Since in the present study, the concentration of PVP is maintained constant (i.e., the number of monomers introduced in the medium is unchanged, only the molecular weight of the macromolecular chains is varied), the retainment of cubic habit was expected. However, regarding the last point, the scientific literature reported that long chains of PVP provide enhanced stability, whereas there are conflicting studies about the influence of the molecular weight of PVP on its reducing ability.^79,80^ In particular, Rashid et al.^79^ in their study on the synthesis of Ag nanoparticles employing PVP with different molecular weights demonstrate that there is no well-defined trend in the size of nanoparticles with change in the PVP molecular weight, except by working at low concentrations (namely, 1 wt.%), where it was registered an increase of the nanoparticles’ size with the increase in the PVP molecular weight. Vice versa, at higher concentrations, a random trend was observed. Moreover, the structural XRPD analysis revealed only the presence of bare Cu_2_O crystal phase (Figure S11).

#### 2.2.4 AA Aqueous Solution: Concentration and Time

To verify the effect of varying the amount of reducing agent, different syntheses were performed varying the AA concentration, ranging from 0 to 2.64 M. Morphology characterization is summarized in Figure 14, whereas the structural XRPD analysis is reported in Figure 15. In the absence of the reducing agent AA (PA-9 sample; Figure 14a), a large flake-like morphology was registered (ca. 1.48 ± 0.70 μm), whereas the structural analysis revealed the presence of signals at 2θ = 32.5° (110), 35.6° (−111), 38.8° (111), 46.3° (−112), 48.7° (−202), 53.5° (020), 58.4° (202), 61.6° (−113), 66.3° (−311), 68.2° (220), 72.5° (311), and 75.3° (−222) (CuO, 01–085–1326; Figure 15), ascribable to the presence of pure CuO crystal flakes (a habit frequently observed in natural samples), thus indicating the retainment of the Cu­(II) oxidation state.^44^ Vice versa, by doubling the amount of the reducing agent AA from 1.34 M (PA-1; Figure 14b) to 2.64 M (PA-10; Figure 14c), cubic particles were obtained. The XRPD analysis revealed the presence of Cu_2_O crystal phase signals in both cases (Figure 15). Macroscopically, an optical transition from brick-orange (PA-1) to yellow (PA-10) was observed, associated to the band gap variation induced by the particles’ size variation.^81^ SEM micrographs confirmed the decrease of the average particle size by doubling the amount of reducing agent, from ca. 349 ± 165 nm (PA-1) to ca. 150 ± 56 nm (PA-10), probably due to the supersaturation of the medium, which pushes the nucleation rate. This trend agrees with the studies by Asamoah et al.^82^ and de Sa et al.,^83^ where it has been demonstrated that to produce finer Ag nanoparticles the concentration of the reducing agent should be in large excess respect to the metal precursor.

**Figure 14:**
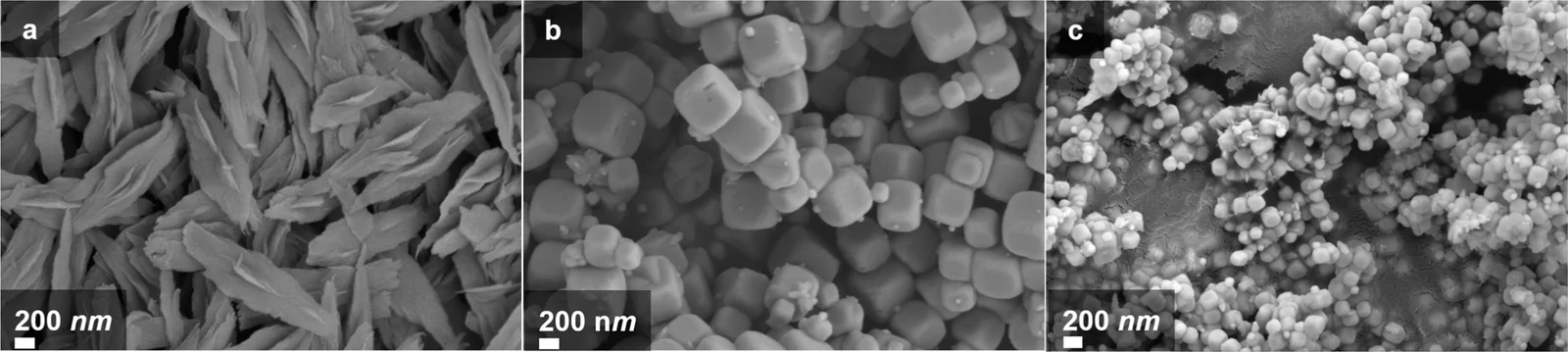
SEM micrographs of PA samples: effect of the concentration of the AA aqueous solution, namely: (a) PA-9 (0.00 M [AA]), (b) PA-1 (1.32 M [AA]), and (c) PA-10 (2.64 M [AA]). All micrographs were collected at the same magnification (50k ×).

**Figure 15:**
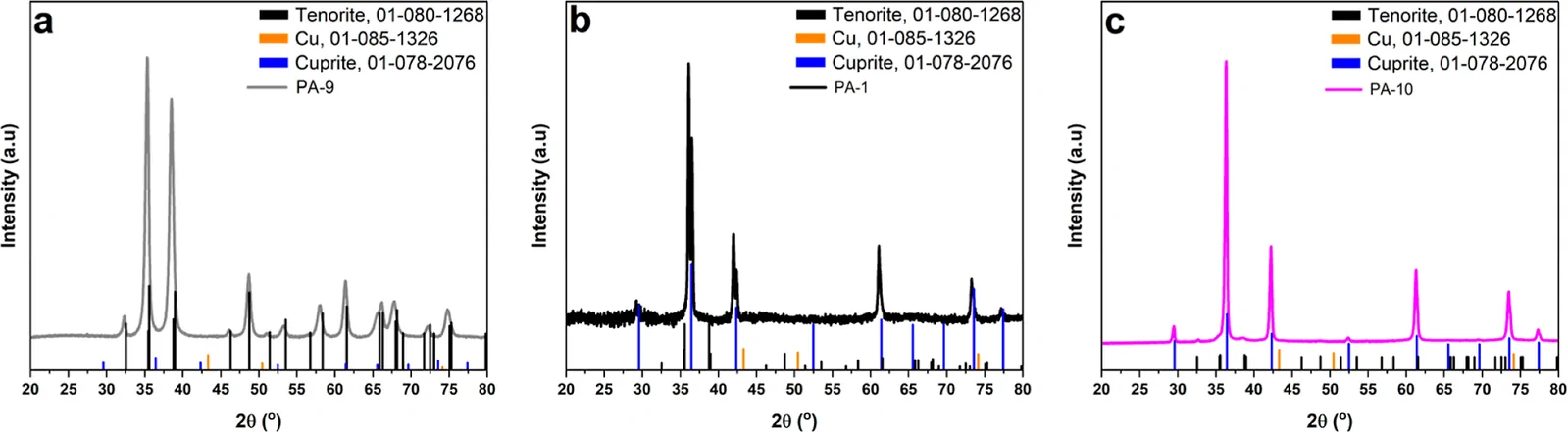
XRPD patterns of PA samples: the concentration of the AA aqueous solution, namely: (a) PA-9 (0.00 M [AA]; gray), (b) PA-1 (1.32 M [AA]; black), and (c) PA-10 (2.64 M [AA]; magenta). XRPD reference patterns: metallic Cu (Cu, 01-085-1326, orange), cuprite (Cu_2_O, 01–078–2076, blue), and tenorite (CuO, 01-080-1268, black).

Lastly, in order to unveil the effect of the reaction time, a further test was performed introducing 10 min of extra time between the precipitating agent NaOH addition and the introduction of the reducing agent AA solution, thus allowing a better homogenization of the reaction solution and providing a better control over the precipitation step. Both SEM micrographs and relative XRPD analysis are reported in the Supporting Information (i.e., Figures S12, and S13, respectively). Results indicated that this modification to the synthesis procedure affected the average particle size. In fact, by increasing the waiting time between the addition of NaOH and the AA, the average particle size decreases significantly, namely: from ca. 349 ± 165 nm (PA-1) to ca. 194 ± 77 nm (PA-11). Additionally, SEM images of PA-11 sample show the growth of tiny, elongated crystals on the surface of the cubes, probably due to the formation of tenorite crystals. XRPD analysis confirmed the presence of Cu_2_O crystal phase as a principal phase coupled with a significant presence of CuO crystal phase (the latter phase confirmed by the presence of the main typical signals).

### 2.3 Morphology Variations Induced by Replacing Glucose with PVP/AA

In order to clarify the role played by the capping/reducing agents, further syntheses were performed by properly modifying the glucose-mediated protocol by replacing glucose with PVP and/or AA, thus trying to convert the single-component glucose-mediated route into the bicomponent PVP/AA-mediated protocol. This way, it was possible to monitor the behavior and potentiality of both PVP and AA employing different synthesis conditions. More specifically, starting from the selected glucose-mediated synthesis labeled Glu-1 (i.e., employing 1.8 g of glucose and able to obtain large octahedral cuprite particles of ca. 3.57 ± 0.43 μm size presenting rounded edges), three further samples were prepared, namely: (i) PvG-1 obtained by replacing glucose with the same amount of PVP-10k (1.8 g), (ii) AvG-1 obtained by replacing glucose with the same amount of AA (1.8 g, 10.0 mmol), and (iii) PAvG-1 obtained by replacing glucose with both PVP-10k and AA while maintaining the PVP-10k:AA molar ratio as in PA-1 (1.7 g of AA, and 0.7 g of PVP-10k; total amount ca. 1.8 g). These comparative substitution experiments were designed to identify the qualitative roles of glucose, PVP, and AA within the synthetic pathway rather than to compare chemically equivalent functional group concentrations. Since these additives differ substantially in molecular architecture, reducing power, adsorption affinity, and steric effects, exerting a direct molar equivalence of hydroxyl or pyrrolidone groups would not represent equivalent reaction conditions, thus in the present study equivalent weight substitution were investigated. Figure 16 reports the SEM micrographs of particles obtained by replacing glucose with PVP/AA, whereas the relative XRPD analysis is reported in Figure 17.

**Figure 16:**
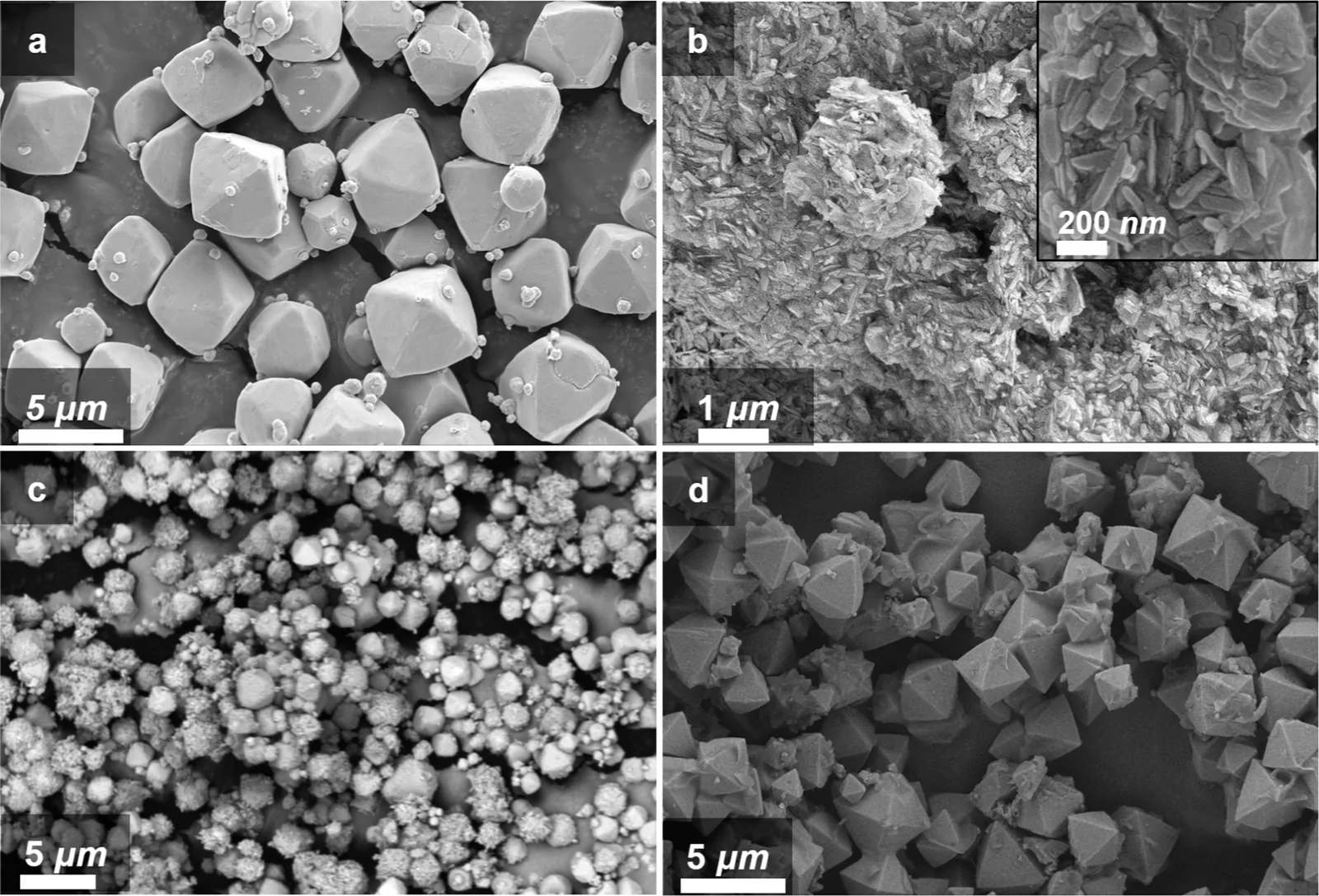
SEM micrographs of samples obtained by replacing glucose with PVP/AA: effect of the capping/reducing agent, namely: (a) Glu-1 (Glu 1.8 g), (b) PvG-1 (PVP 1.8 g), (c) AvG-1 (AA 1.8 g), and (d) PAvG-1 (PVP 0.7 g, AA 1.7 g). All micrographs were collected at the same magnification (10k ×), with the exception of panel (b) which was collected at high magnification (30k ×) and inset in panel (b) which was collected at very high magnification (100k ×).

**Figure 17:**
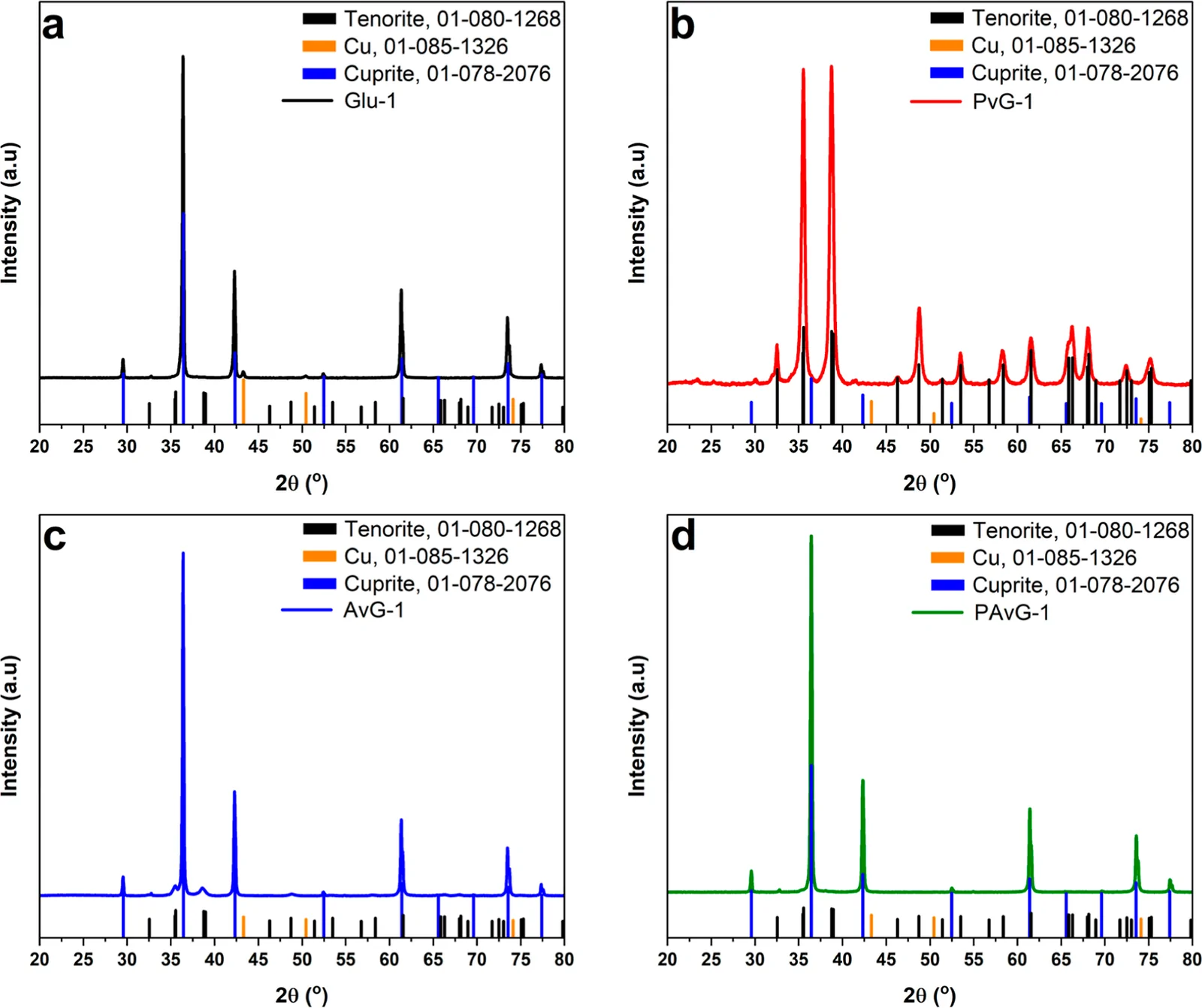
XRPD patterns of samples obtained by replacing glucose with PVP/AA: effect of the capping/reducing agent, namely: (a) Glu-1 (Glu 1.8 g; black), (b) PvG-1 (PVP 1.8 g; red), (c) AvG-1 (AA 1.8 g; blue), and (d) PAvG-1 (PVP 0.7 g, AA 1.7 g; green). XRPD reference patterns: metallic Cu (Cu, 01-085-1326, orange), cuprite (Cu_2_O, 01-078-2076, blue), and tenorite (CuO, 01-080-1268, black).

From the morphological viewpoint, the PvG-1 sample consists of aggregates of flake-like particles of ca. 285 ± 78 nm, forming structures resembling desert roses (Figure 16b). As expected, the XRPD analysis revealed the presence of only CuO crystal phase: this result is not surprising since no reducing agent was added to the reaction medium. Vice versa, in the case of AvG-1 sample obtained employing AA instead of glucose and in absence of any capping agent (i.e., PVP), the morphology of the sample looks constituted by octahedral particles (ca. 1.40 ± 0.58 μm) covered with tiny crystals (Figure 16c). The XRPD analysis of AvG-1 sample revealed the presence of signals of the cuprite Cu_2_O crystal phase, surprisingly with traces of CuO crystal phase. On the contrary, SEM micrograph of the PAvG-1 sample obtained by replacing glucose with both the capping agent PVP and the reducing agent AA results primarily being made by octahedral particles, with very slightly round edges and average particle size of ca. 1.50 ± 0.60 μm, thus remarkably smaller than in the case of the glucose-mediated system (ca. 3.57 ± 0.43 μm, Glu-1; Figure 16a). As expected, the XRPD analysis of PAvG-1 revealed the presence of signals due to the cuprite Cu_2_O crystal phase.

Since glucose and PVP/AA were employed to investigate different chemical functions within the crystal growth process, their concentrations were not normalized according to the molar amount of either hydroxyl or pyrrolidone functional groups. In fact, such normalization would not provide chemically equivalent conditions, since glucose acts both as a reducing/capping agent, whereas PVP/AA combines a reducing agent (AA) with a polymeric capping agent (PVP). The comparison was therefore designed to distinguish the effects of the different chemical components on Cu_2_O formation and crystal growth rather than to establish a stoichiometric equivalence among their functional groups.

The comparison between PvG-1 and AvG-1 clearly demonstrates that the formation of Cu_2_O is controlled primarily by the presence of an effective reducing agent (AA) rather than by the presence of PVP. While AA readily reduces the Cu­(II) intermediate to Cu­(I), PVP alone cannot alter the copper oxidation state under these strongly alkaline conditions and therefore mainly acts as a surface-adsorbing polymer (capping agent). Consequently, PVP influences only crystal nucleation and growth, whereas the reduction step is governed by either glucose or AA.

Interestingly, these further case studies demonstrated that the capping agent PVP, under different synthesis conditions, and with the support of the reducing agent AA, allows the formation of not only cubic (PA-4; Figure 7a) and spheroidal (PA-2; Figure 7d) cuprite Cu_2_O particles but also octahedral (PAvG-1; Figure 16d) particles, thus attesting the PVP/AA bicomponent system as an extremely powerful system in finely shaping cuprite Cu_2_O particles.

Lastly, FTIR spectra were recorded to investigate the possible interaction between the Cu_2_O particles and the capping/reducing agents employed during the synthesis, namely glucose, PVP and/or AA. Figure S14 reports a comparison between the Glu-1 sample and the samples obtained by replacing glucose with PVP and/or AA (i.e., PvG-1, AvG-1, and PAvG-1 samples), together with the spectra of the corresponding reference compounds (glucose, PVP, and AA). The FTIR spectrum of the reference Cu_2_O exhibits only one absorption band at ca. 624 cm^–1^, which can be attributed to the Cu­(I)–O vibrational mode.^84^ In contrast, the spectrum of Glu-1 shows an additional contribution at ca. 1100 cm^–1^, consistent with the skeletal ring vibrations of glucose/gluconate species.^85^ The PvG-1 sample also exhibits bands at ca. 1650 cm^–1^_,_ 1460 cm^–1^, and 1290 cm^–1^, which can be assigned to characteristic vibrational modes of PVP, including C = O stretching, C–H bending, and contributions from C–H wagging and C–N stretching vibrations, respectively,^86,87^ together with the absence of the cuprite characteristic signal at ca. 624 cm^–1^, thus confirming the formation of the tenorite crystal phase. Similarly, the FTIR spectrum of AvG-1 shows a contribution at ca. 1100 cm^–1^, attributable to C–O stretching, together with weaker bands at ca. 3400 cm^–1^ and at 1654 cm^–1^, assigned to O–H stretching and C=C stretching-related vibrations, respectively, supporting the presence of AA.^88^ Interestingly, the PAvG-1 sample exhibits the simultaneous presence of additional signals at ca. 3400 cm^–1^, 1654 cm^–1^, and 1100 cm^–1^, consistent with the coexistence of PVP and AA species sorbed at the cuprite surface.

These FTIR results provide evidence for the presence of glucose/gluconate, PVP, and AA species associated with the surface of the ex situ Cu_2_O particles. However, the detection of these organic species after isolation and washing of the Cu_2_O crystals does not demonstrate that they acted as structure-directing agents during crystal growth. Rather, the FTIR data provide evidence of the ability of these molecules (or their oxidation products) to interact with and/or remain associated with the Cu_2_O surface. Such interactions are consistent with the possible involvement of these species in modifying crystal growth during synthesis; although the FTIR measurements performed on the ex-situ products do not allow the specific adsorption sites, adsorption geometry, or facet selectivity to be established. Finally, it must be remembered that foreign molecules can be incorporated into the product’s crystal structure due to growth mechanisms, e.g. impurities or additives but even polymeric gel networks can be found within the crystal’s volume.^89–91^ Incidentally, the paper by Gavira et al.^90^ shows protein single crystals which become highly rounded as a collateral effect of the presence of the gel network.

## 3 Conclusions

The present work provides a systematic investigation of the influence of synthesis parameters on the morphology and crystal growth of Cu_2_O particles obtained through two wet-chemical precipitation routes based on either glucose or a PVP/ascorbic acid (PVP/AA). The results demonstrate that both the particle size and crystal habit are strongly dependent on the chemical environment established during synthesis and on the relative contributions of the reducing and surface-interacting species.

In the glucose-mediated route, variations in the hydroxide and glucose amounts, reaction volume, temperature, and copper precursor resulted in significant changes in particle size and morphology. Depending on the synthesis conditions, octahedral, truncated octahedral, cubic, and more complex polyhedral morphologies were obtained, exposing different combinations of {100}, {110}, {111}, and high-index facets. In particular, the amount of NaOH and the glucose concentration were found to be the most influential parameters. Decreasing the amount of NaOH resulted in smaller particles and promoted a greater contribution of the {110} facets, whereas changes in the glucose concentration mainly affected the relative development of the {100} and {111} facets. The reaction volume and temperature also affected the final morphology and particle dimensions, highlighting the sensitivity of glucose-mediated synthesis to the overall reaction conditions. The nature of the copper precursor and the order of reagent addition further demonstrated that changes in the precursor speciation and reaction pathway can significantly influence phase formation and crystal growth.

The PVP/AA-mediated route exhibited distinct morphological evolution. In this system, the PVP concentration was the parameter with the most pronounced effect on crystal morphology. Increasing the PVP concentration promoted the evolution from predominantly cubic particles toward progressively more rounded particles, while crystallographic analysis indicated that the apparently spheroidal particles are constituted by highly faceted crystals, presenting high crystallographic coherence, bounded by numerous high-index surfaces. The comparison with the glucose route highlights the importance of separating the reducing and surface-interacting functions: whereas glucose simultaneously contributes to the reduction process and modifies the growth environment, in the PVP/AA system, AA acts as the reducing agent while PVP provides the polymeric surface-interacting component.

Overall, the results establish clear relationships between synthesis parameters and Cu_2_O crystal morphology, demonstrating that the formation of selected crystal habits and the development of different crystallographic surfaces can be directed through the appropriate control of solution composition and reaction conditions. The systematic correlations established here, together with a crystal growth interpretation based on additive-mediated stabilization of high-index facets, provide a mechanistic basis for the rational design of Cu_2_O crystals with tailored exposed facets and complex morphologies. In addition to offering valuable guidelines for optimizing copper oxide materials in applications where surface structure plays a critical role (including electrocatalysis, photocatalysis, and sensing), the mechanistic insights gained in this work may also contribute to the rational design of other well-structured oxide materials synthesized through wet-chemical routes.

## 4 Experimental Section

### 4.1 Chemicals

Inorganic salts: copper­(II) acetate monohydrate (Cu­(CH_3_COO)_2_·H_2_O, 99.2%, CAS 6046-93-1, Sigma-Aldrich), copper­(II) nitrate trihydrate (Cu­(NO_3_)_2_·3H_2_O, 99–104%, CAS 10031-43-3, Sigma-Aldrich), copper­(II) sulfate pentahydrate (CuSO_4_·5H_2_O, 98%, CAS 23254-43-5, Sigma-Aldrich), reference copper­(I) oxide (Cu_2_O, 99.9% (metal basis), CAS 1317-39-1, Thermo scientific), and sodium hydroxide pellets (NaOH, 99%, 1310-73-2, VWR), are used as received. Organic compounds: polyvinylpyrrolidone average molecular weight 10,000 (PVP-10k, CAS 9003-39-8, Sigma-Aldrich), polyvinylpyrrolidone average molecular weight 55,000 (PVP-55k, CAS 9003-39-8, Sigma-Aldrich), ascorbic acid (AA, C_6_H_8_O_6_, 99+%, CAS 50-81-7, Thermo-Fisher), and glucose (Glu, C_6_H_12_O_6_, 99.5%, CAS 50-99-7, Sigma-Aldrich), are used as received. Both deionized water and ethanol (C_2_H_6_O, 99.8%, CAS 64-17-5, Honeywell) are used during washing procedures. Milli-Q water with a resistivity of 18.2 MΩ cm is used. All chemicals are used without further purification.

### 4.2 Synthesis of Cuprite Particles Employing Glucose as a Capping/Reducing Agent

Cuprite particles are prepared following a modified procedure taken from the literature.^92^ In this protocol, copper­(II) salts (i.e., copper­(II) sulfate pentahydrate, copper­(II) acetate monohydrate, and copper­(II) nitrate trihydrate) are used as cuprite precursors, NaOH as a precipitating agent, and glucose as a capping/reducing agent. In a typical synthesis, copper­(II) sulfate pentahydrate (5.00 g, 20.0 mmol) is dissolved in a round-bottomed flask containing a fixed volume of deionized water (100 mL) at 70 °C, thus obtaining a light-blue solution. Subsequently, after 5 min, NaOH pellets (9.6 g; 240.0 mmol) are added to the solution. With the dissolution of the NaOH pellets (which takes ca. 5 min), the formation of a black CuO precipitate occurs. Lastly, glucose powders (1.8 g; 10.0 mmol) are added to the suspension (i.e., final volume: 100 mL), observing a variation of the color of the precipitate (i.e., from black to red), thus registering the formation of cuprite particles. The system is maintained under mixing for 10 min, then solid particles are recovered by centrifugation (9000 rpm, 4 min), and purified by washing with deionized water (3×) and ethanol (1×). Finally, cuprite particles are oven-dried at 75 °C for 24 h under an air atmosphere and kept in a close glass vial at RT in the dark. This typical synthesis has been defined as Glu-1, where Glu stands for glucose. Subsequently, the effect of several experimental parameters is investigated by making the following changes:

- Delivery and order of reactants, with prior NaOH and then Glu delivered both as solid powders (Glu-1), to an inverted order (i.e., prior Glu then NaOH; Glu-2) or with NaOH delivered as an aqueous solution (Glu-3).
- The initial volume of deionized water was from 100 mL (Glu-1) to 80 mL (Glu-4), 120 mL (Glu-5), or 200 mL (Glu-6).
- Reaction temperature: from 70 °C (Glu-1) to RT (Glu-7), 50 °C (Glu-8), or 90 °C (Glu-9).
- Quantity of reactants: (i) NaOH (9.6 g): from 1.8 g of Glu (Glu-1) to half/double the amount of glucose (Glu-10/Glu-11) and (ii) NaOH (4.8 g): from 1.8 g of (Glu-12) or half/double the amount of glucose (Glu-13/Glu-14).
- Cu precursors: replacement of copper­(II) sulfate pentahydrate (5.00 g, 20.0 mmol; both Glu-1 and Glu-13) with either copper­(II) nitrate trihydrate (4.84 g, 20.0 mmol; both Glu-15 and Glu-17) or copper­(II) acetate monohydrate (4.00 g, 20.0 mmol; both Glu-16 and Glu-18).

All changes in terms of compositional and experimental conditions are reported in Table 1.

### 4.3 Synthesis of Cuprite Particles Employing PVP/Ascorbic Acid as a Capping/Reducing Agent

Cuprite particles are prepared following a modified procedure taken from the literature.^49^ In this protocol of synthesis, copper­(II) salts (i.e., either copper­(II) acetate monohydrate or copper­(II) nitrate trihydrate) are used as cuprite precursors, NaOH as a precipitating agent, PVP (either PVP-10k or PVP-55k) as a capping agent, and AA as a reducing agent. In a typical synthesis, both copper­(II) acetate monohydrate (0.50 g, 2.50 mmol) and PVP-10k (0.50 g) are dissolved in a round-bottomed flask containing a fixed volume of deionized water (400 mL) at room temperature (RT), thus obtaining a light-blue solution. Afterward, a previously prepared 1.25 M NaOH solution (2.00 g of NaOH in 40 mL of deionized water) is added dropwise (delivery time: 10 min) to the solution, observing the formation of a blue precipitate identified as Cu­(OH)_2_. Subsequently, a previously prepared 1.32 M AA aqueous solution (volume: 60 mL) is added dropwise (delivery time: 10 min) to the suspension (i.e., final volume: 500 mL), observing a rapid variation of the color of the precipitate (i.e., from blue to orange), thus registering the formation of cuprite particles. The system is maintained under mixing for 10 min, then solid particles are recovered by centrifugation (9000 rpm, 4 min), and purified by washing with deionized water (3×) and ethanol (1×). Finally, cuprite particles are oven dried at 75 °C for 24 h and kept in a closed glass vial at RT in the dark. This typical synthesis has been defined as PA-1, where PA stands for PVP and AA, respectively. Subsequently, the effect of several experimental parameters is investigated by making the following changes:

- The initial volume of deionized water was from 400 mL (PA-1) to 100 mL (PA-2), 200 mL (PA-3), or 500 mL (PA-4). This way, both the final volume and the water:NAOH:AA volume ratio are modified, consequently.
- Reaction temperature: from RT (PA-1) to either 50 °C (PA-5) or 75 °C (PA-6).
- Cu­(II) salt: replacement of copper­(II) acetate monohydrate (0.50 g, 2.50 mmol; PA-1) with copper­(II) nitrate trihydrate (0.61 g, 2.50 mmol; PA-7).
- PVP molecular weight: replacement of PVP-10k (0.50 g; PA-1) with PVP-55k (0.50 g; PA-8).
- Concentration of the AA aqueous solution: from 1.32 M (PA-1) to either 0 (PA-9) or 2.64 M (PA-10).
- Time prior to adding the AA solution: either immediately (PA-1) or after waiting for an additional 10 min (PA-11).

All changes in terms of compositional and experimental conditions are reported in Table 2.

**Table 2:** Compositional and Experimental Parameters Adopted during the Synthesis, Structural and Morphology Characterization of PA-Samples, Namely by Employing PVP/AA as a Capping/Reducing Agent^a^

samples	Cu(II) salt	PVP	[AA] (M)	water/NaOH/AA vol. ratio (mL/mL/mL)	temperature (°C)	time prior to add AA solution (min)	XRPD			SEM	
							crystal phase	Cu_2_O lattice parameters *a* = *b* = *c* (Å)	Cu_2_O Scherrer crystallite average size τ (nm)	morphology	average particle size (nm)
**PA-1**	**acetate**	**10k**	**1.32**	**400:40:60**	**RT**	**0**	**Cu**_2_ **O**	**4.29**	**44**	**cube**	349 ± 165
PA-2	acetate	10k	1.32	100:40:60	RT	0	Cu_2_O, amorphous	4.26	26	spherical	550 ± 237
PA-3	acetate	10k	1.32	200:40:60	RT	0	Cu_2_O	4.26	58	rounded cube	447 ± 241
PA-4	acetate	10k	1.32	500:40:60	RT	0	Cu_2_O	4.27	56	cube	283 ± 117
PA-5	acetate	10k	1.32	400:40:60	50	0	Cu_2_O (CuO)	4.27	59	cube	298 ± 134
PA-6	acetate	10k	1.32	400:40:60	75	0	Cu_2_O	4.27	63	cube	290 ± 107
PA-7	nitrate	10k	1.32	400:40:60	RT	0	Cu_2_O, CuO	4.27	54	cube	446 ± 177
PA-8	acetate	55k	1.32	400:40:60	RT	0	Cu_2_O	4.26	28	cube	259 ± 78
PA-9	acetate	10k	0	400:40:60 ^a)^	RT	0	CuO	4.27	17	flake-like	1485 ± 703
PA-10	acetate	10k	2.64	400:40:60	RT	0	Cu_2_O	4.27	30	cube	150 ± 56
PA-11	acetate	10k	1.32	400:40:60	RT	10	Cu_2_O, CuO	4.28	45	cube	194 ± 77

a Since the synthesis has been carried out without adding AA but simply by introducing deionized water, the effective water/NaOH/AA vol. ratio is 460:40:0.

### 4.4 Synthesis of Cuprite Particles Employing PVP/Ascorbic Acid Substituting Glucose as a Capping/Reducing Agent

In order to unveil the role played by capping/reducing agents employed in the previous protocols (i.e., either glucose or PVP/AA), cuprite particles are also prepared by properly modifying the glucose-mediated protocol (presented in Section 4.2) by replacing glucose with PVP and/or AA, trying to convert the single-component glucose-mediated route into the bicomponent PVP/AA-mediated protocol (presented in Section 4.3). This way, it is possible to monitor the behavior of both PVP and AA in another formulation. The selected glucose-mediated formulation is the one labeled Glu-1. In a typical synthesis, copper­(II) sulfate pentahydrate (5.00 g, 20.0 mmol) is dissolved in a round-bottomed flask containing a fixed volume of deionized water (100 mL) at 70 °C, thus obtaining a light-blue solution. Subsequently, after 5 min NaOH pellets (9.6 g; 240.0 mmol) are added to the solution. With the dissolution of the NaOH pellets (which takes ca. 5 min), the formation of a black precipitate occurs. Lastly, capping/reducing agents are added to the suspension (i.e., final volume: 100 mL). In particular, three case studies were monitored, namely: (i) the glucose replacement with PVP-10k (1.8 g; PvG-1), (ii) the glucose replacement with AA (1.8 g, 10.0 mmol; AvG-1), and (iii) the glucose replacement with both PVP-10k and AA maintaining the PVP-10k:AA molar ratio as in PA-1 (1.7 g of AA, and 0.7 g of PVP-10k; PAvG-1). The system is maintained under mixing for 10 min, then solid particles are recovered by centrifugation (9000 rpm, 4 min) and purified by washing with deionized water (3×) and ethanol (1×). Finally, cuprite particles are oven dried at 75 °C for 24 h and kept in a close glass vial at RT in the dark. All changes in terms of compositional and experimental conditions are reported in Table 3.

**Table 3:** Compositional and Experimental Parameters Adopted during the Synthesis, Structural and Morphology Characterization of Glu-1 Formulation by Replacing Glucose with PVP and/or AA as a Capping/Reducing Agent

samples	Cu(II) salt	NaOH (g)	Glu (g)	PVP-10k (g)	AA (g)	water volume (mL)	XRPD			SEM	
							crystal phase	Cu_2_O lattice parameters *a* = *b* = *c* (Å)	Cu_2_O Scherrer crystallite average size τ (nm)	morphology	average particle size (nm)
**Glu-1**	**sulfate**	**9.6**	**1.8**	**0.0**	**0.0**	**100**	**Cu**_2_ **O (Cu)**	**4.27**	**68**	**octahedral**	3573 ± 432
PvG-1	sulfate	9.6	0.0	1.8	0.0	100	CuO	4.26	21	lamellar	285 ± 78
AvG-1	sulfate	9.6	0.0	0.0	1.8	100	Cu_2_O (CuO)	4.27	68	octahedral covered with tiny crystals	1398 ± 578
PAvG-1	sulfate	9.6	0.0	0.7	1.7	100	Cu_2_O	4.26	58	octahedral	1499 ± 604

### 4.5 Morphological and Structural Characterization

High magnifications scanning electron microscopy (SEM) micrographs were obtained using a Zeiss Gemini 500 microscope equipped with a field-emission source (FEG) and a Bruker Quantax detector for energy-dispersive X-ray spectroscopy (EDS) microanalysis. The microscope was operated at an acceleration voltage of 5.00 kV. Micrographs were captured using the “in-lens” detector. Low magnifications SEM micrographs, instead, were obtained using a Thermo Scientific Phenom G6 Desktop microscope operating at accelerating voltages of 5.00 kV. Micrographs were captured using the backscattered electron detector (BSD). In both cases, samples were deposited onto SEM stubs using a double-adhesive carbon tape, and then they were covered with Au coating to prevent charging effects using a VPI SD-900C sputter-coater (sputtering time: 40 s). The dimensional statistical analysis was performed using the software ImageJ, analyzing at least 50 particles per micrograph.

Transmission electron microscopy (TEM) imaging and selected area electron diffraction (SAED) analyses were performed with a TEM JEOL JEM 2100 Plus working with a LaB_6_ emitter and an electron gun operating at 200 kV. The microscope used is equipped with an 8-megapixel Gatan (Gatan, USA) Rio complementary metal-oxide-semiconductor (CMOS) camera. Samples were deposited onto carbon-coated Cu TEM mesh grids by drop-casting a dilute dispersion of cuprite particles in acetone.

X-ray powder diffraction (XRPD) patterns were recorded using a Rigaku Miniflex 600 diffractometer with a Cu source working at 40 kV and 15 mA, scanning in a 2θ range of 10–80° with a step of 0.02°, and an angular velocity of 5.0°/min, D/teX Si detector. XRD patterns were indexed according to the ICDD database using the instrumental PDXL-2 software. The crystallite average size (τ, expressed in nm) was estimated by means of the Scherrer equation. For the cuprite (Cu_2_O) crystal phase, the Bragg angle selected is the crystal reflection at 2θ ca. 36.4°, corresponding to the Miller index (111). The lattice parameters were calculated using the instrumental PDXL-2 software.

Crystal models were produced with the program KristalShaper, S. Webber, JCrystalSoft, version 1.4.0.

Structural models of Cu_2_O were made with the program Mercury.^93^

FTIR spectra were recorded in transmission mode on KBr pellets by means of a Jasco-4100 spectrophotometer, equipped with DTGS detector, applying the Blackman-Harris apodization function (4 cm^–1^ resolution, 4000–500 cm^–1^ spectral range, 128 scans). Samples are analyzed after background subtraction performed on a bare KBr pellet.

## Supplementary Material



## Data Availability

Data will be made available on request.

## Notes

Glossary/notations, XRPD patterns of all materials, SEM micrographs of PA-11 material, cuprite crystal morphologies from natural crystals and the proposed morphodrome, FTIR spectra (PDF)
