# Supplementary material for: Chemical Control of Cuprite Crystal Growth and Morphology toward High-Index Surfaces
Source: Inorg Chem. 2026 Oct 10;65(42):24848–70. doi: 10.1021/acs.inorgchem.6c03779 (PMC13715583; doi:10.1021/acs.inorgchem.6c03779)
Supplement: Supplementary file 1 [file ic6c03779_si_001.pdf]

# Chemical Control of Cuprite Crystal Growth and Morphology toward High-Index Surfaces

Gaia Novo, Falak Shafiq, Tatiana Rodriguez-Flores, Roberto Scotti, Massimo Moret,* Roberto Nisticò *

Department of Materials Science, University of Milano-Bicocca, U5, INSTM, Via R. Cozzi 55, 20125 Milano, Italy.
*Corresponding authors. E-mails: massimo.moret@unimib.it (M.M.), roberto.nistico@unimib.it (R.N.).

## Supporting Information

**Glossary/notations** (see also the Online Dictionary of Crystallography at www.iucr.org):

*Form:* a set of faces related to one another by the symmetry elements of the space group/crystal class. A form is identified by the symbol {*hkl*} while (*hkl*) indicates the specific plane with Miller indices *h*, *k*, *l*. The interplanar distance for a family of planes {*hkl*} is $d_{hkl}$.

*Habit:* the crystal habit is the overall external shape of a crystal determined by the actual development of its forms. Based on the development of its faces, a crystal can be further classified as *euhedral* (the faces are well developed due to unconstrained growth), *subhedral* (faces are only partially developed), *anhedral* (the faces are not developed, and Miller indices cannot be attributed without ambiguity). Examples of crystal habits are: whisker, needle, platy, block, prismatic, pyramidal, equant, cubic, octahedral, etc.

*Morphology:* the set of {*hkl*} crystal forms appearing in a crystal. In ideal crystals all faces belonging to a given form have the same development and morphological importance.

*Zone axis:* a lattice row parallel to the intersection of two or more families of lattices planes (or crystal faces). Being a crystallographic direction is denoted with direction indices [*uvw*]. The symbol ⟨*uvw*⟩ indicates a whole family of symmetry related directions.

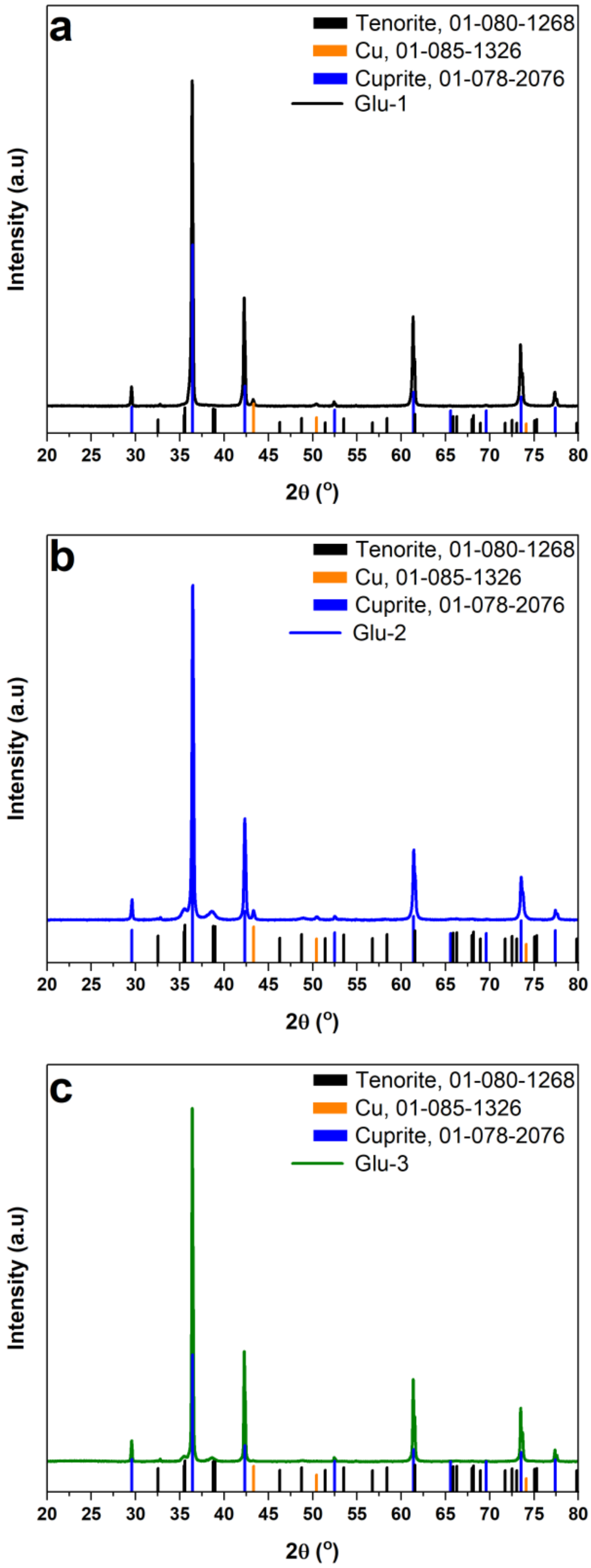


**Figure S1**. XRPD patterns of Glu-samples: effect of delivery and order or reactants, namely (a) Glu-1 (NaOH in pellets; NaOH, Glu; black), (b) Glu-2 (NaOH in pellets; Glu, NaOH; blue), and (b) Glu-3 (addition of a 12 M NaOH solution; NaOH, Glu; green). XRPD reference patterns: metallic Cu (Cu, 01-085-1326, orange), cuprite ($Cu_2O$, 01-078-2076, blue), and tenorite (CuO, 01-080-1268, black).

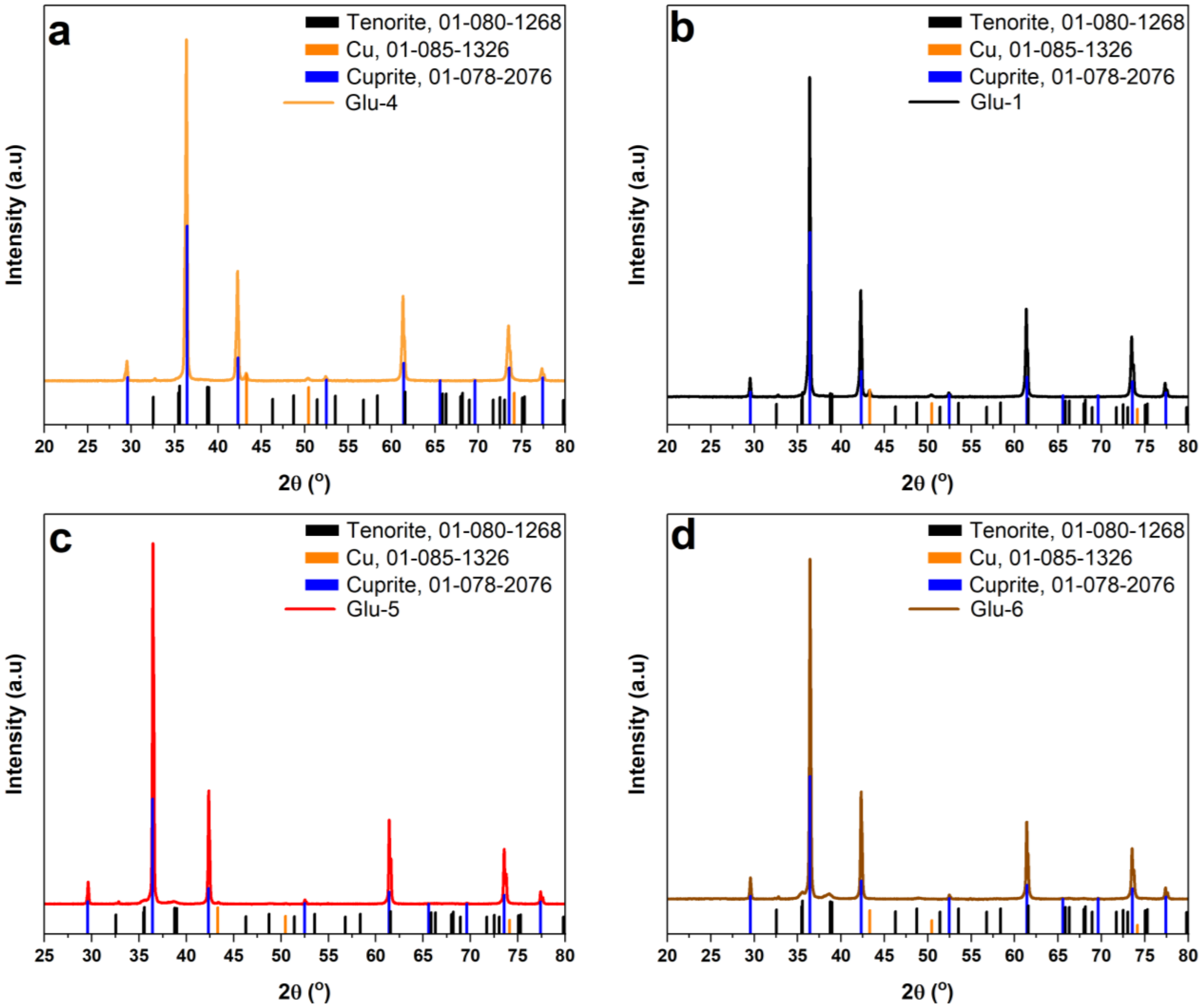


**Figure S2**. XRPD patterns of Glu-samples: effect of the initial volume of deionized water, namely (a) Glu-4 (80 mL; dark yellow), (b) Glu-1 (100 mL; black), (c) Glu-5 (120 mL; red), and (d) Glu-6 (200 mL; brown). XRPD reference patterns: metallic Cu (Cu, 01-085-1326, orange), cuprite ($Cu_2O$, 01-078-2076, blue), and tenorite (CuO, 01-080-1268, black).

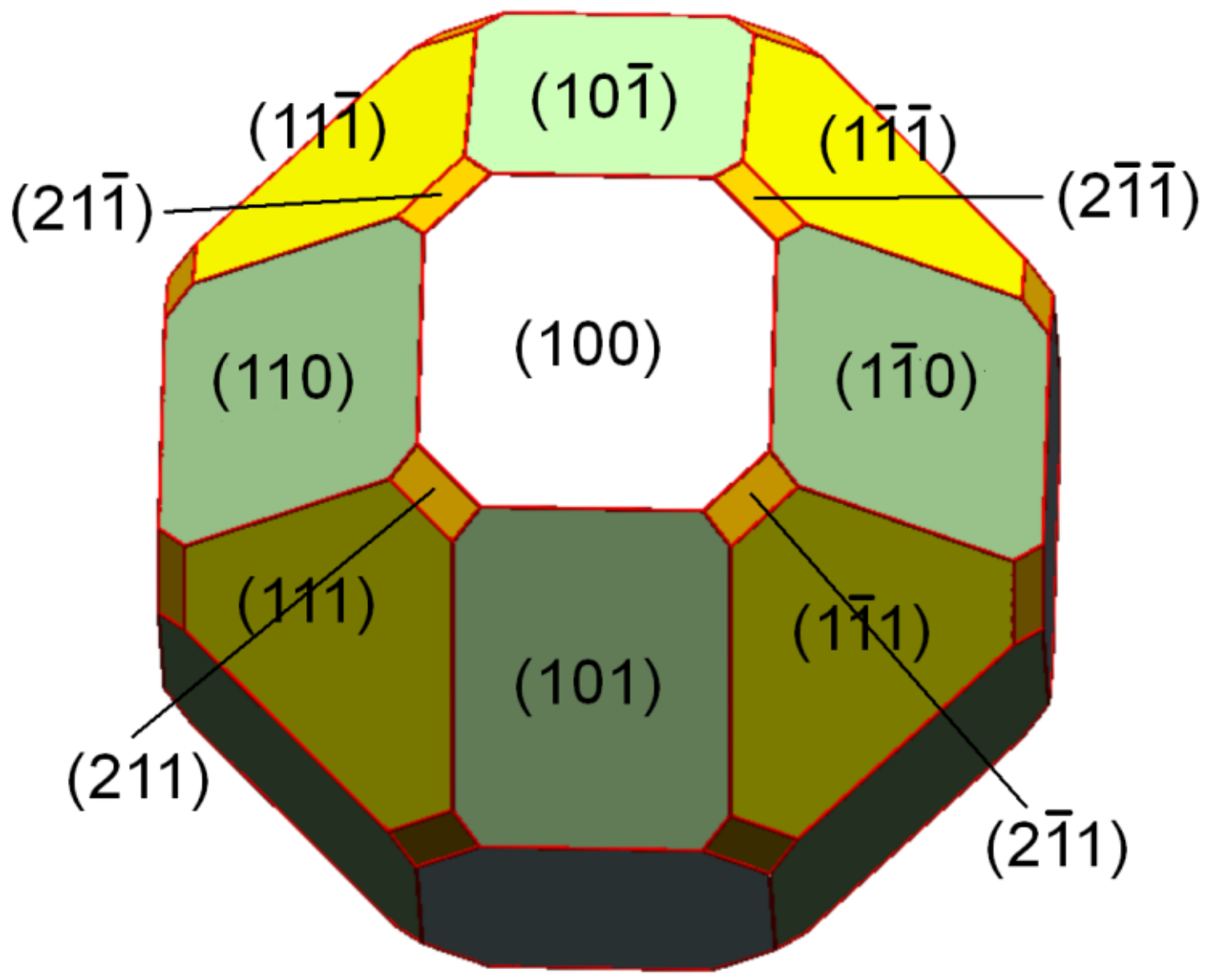


**Figure S3.** Average morphology of crystals in sample Glu-18. Beyond the usual cube, octahedron and rhombic dodecahedron also {211} facets belong to the growth morphology.

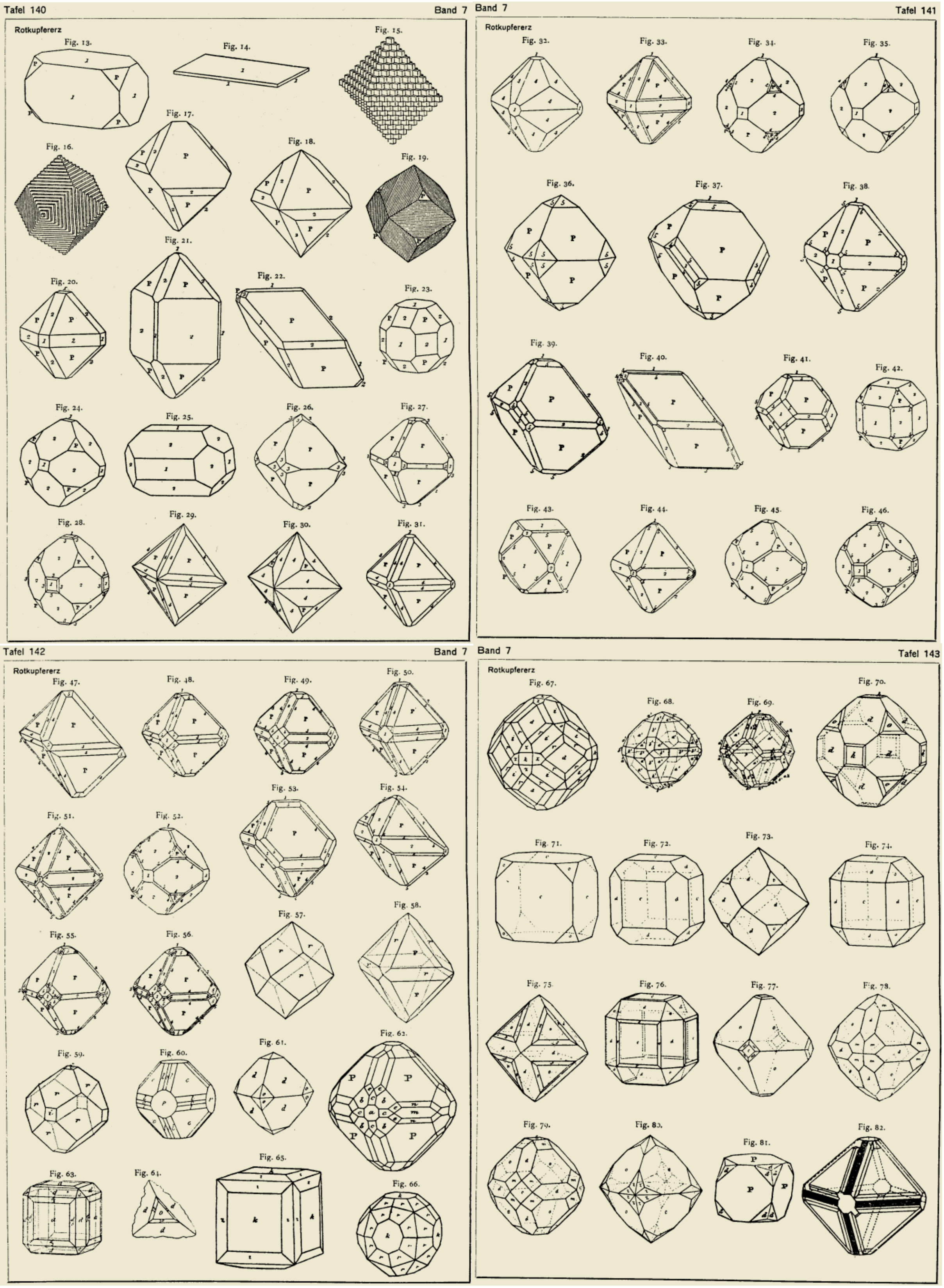


**Figure S4.** A selection of crystal morphologies observed in natural cuprite crystals from *Atlas der Kristallformen*.[1]

(1) Goldschmidt, V. M. Atlas der Kristallformen. Carl Winters Universitatsbuchhandlung, Heidelberg (Germany), **1913-1923**.

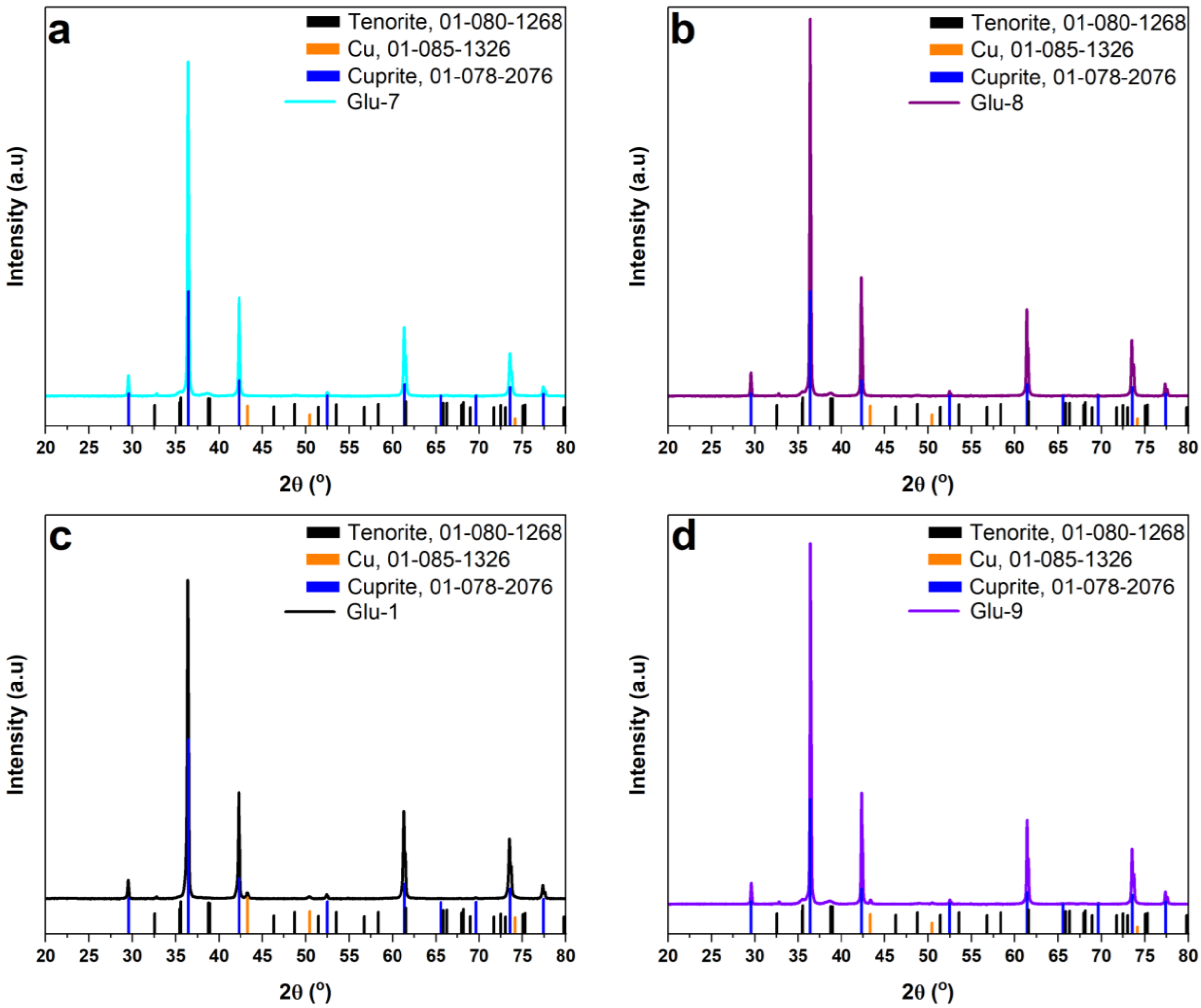


**Figure S5**. XRPD patterns of Glu-samples: effect of the reaction temperature, namely (a) Glu-7 (RT; cyan), (b) Glu-8 (50 °C; purple), (c) Glu-1 (70 °C; black), and (d) Glu-9 (90 °C; violet). XRPD reference patterns: metallic Cu (Cu, 01-085-1326, orange), cuprite ($Cu_2O$, 01-078-2076, blue), and tenorite (CuO, 01-080-1268, black).

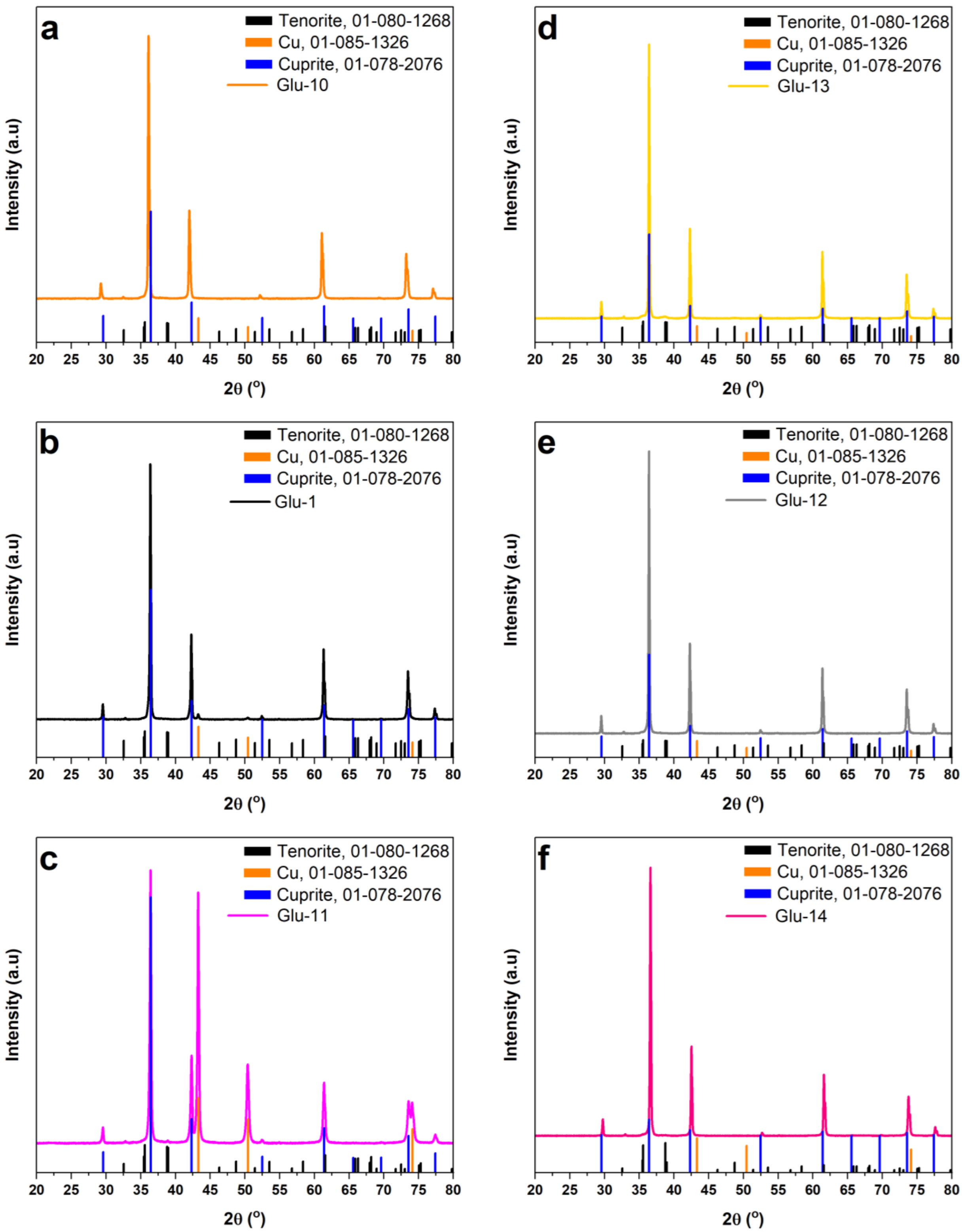


**Figure S6**. XRPD patterns of Glu-samples: effect of the quantity of reactants, namely (a) Glu-10 (NaOH 9.6 g, Glu 0.9 g; orange), (b) Glu-1 (NaOH 9.6 g, Glu 1.8 g; black), (c) Glu-11 (NaOH 9.6 g, Glu 3.6 g; magenta), (d) Glu-13 (NaOH 4.8 g, Glu 0.9 g; yellow), (e) Glu-12 (NaOH 4.8, Glu 1.8 g; grey), and (f) Glu-14 (NaOH 4.8, Glu 3.6 g; pink). XRPD reference patterns: metallic Cu (Cu, 01-085-1326, orange), cuprite ($Cu_2O$, 01-078-2076, blue), and tenorite (CuO, 01-080-1268, black).

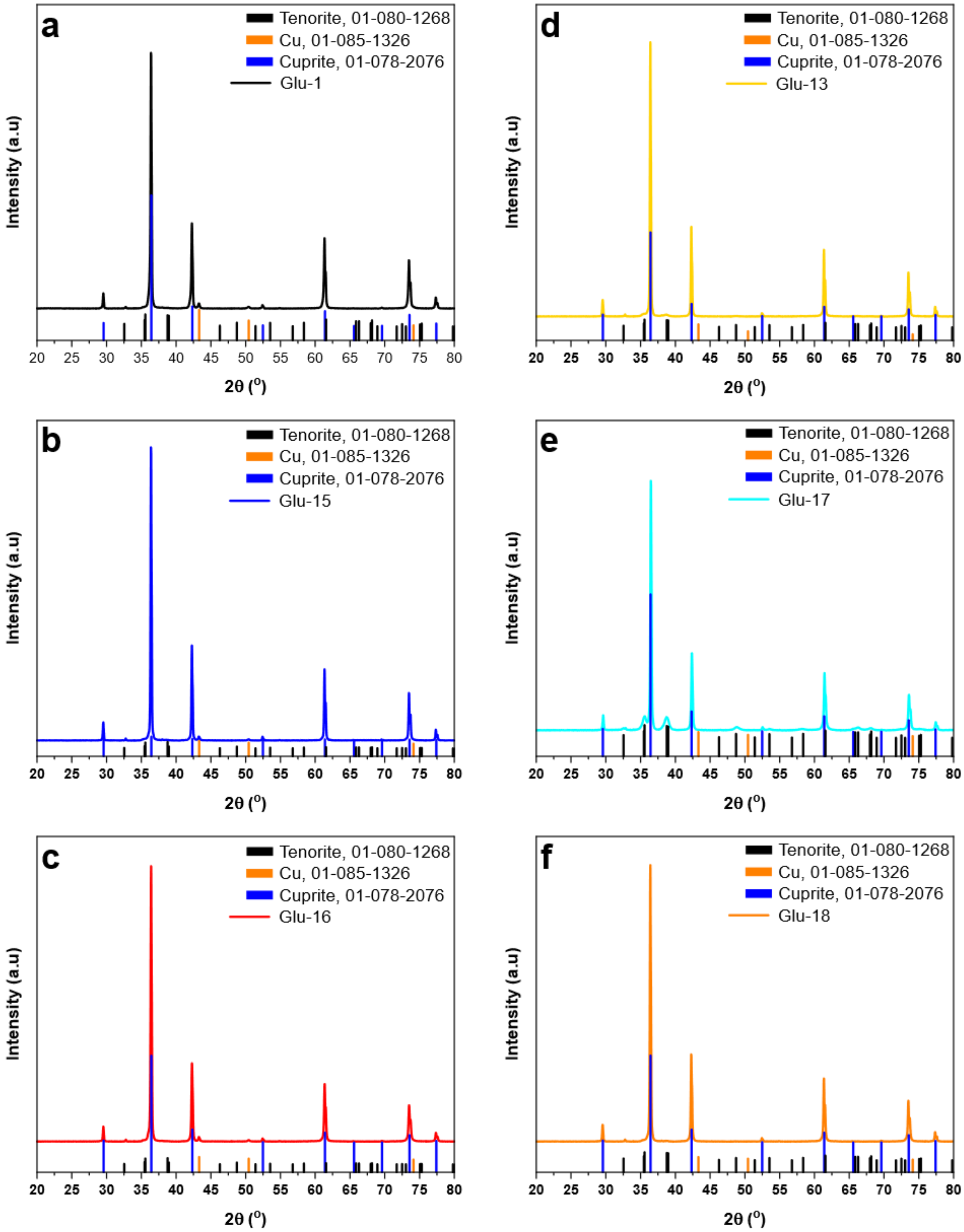


**Figure S7**. XRPD patterns of Glu-samples: effect of the Cu precursors, namely: (a) Glu-1 (Sulfate, NaOH 9.6 g, Glu 1.8 g; black), (b) Glu-15 (Nitrate, NaOH 9.6 g, Glu 1.8 g; blue), (c) Glu-16 (Acetate, NaOH 9.6 g, Glu 1.8 g; red), (d) Glu-13 (Sulfate, NaOH 4.8 g, Glu 0.9 g; yellow), (e) Glu-17 (Nitrate, NaOH 4.8, Glu 0.9 g; cyan), and (f) Glu-18 (Acetate, NaOH 4.8, Glu 0.9 g; orange). XRPD reference patterns: metallic Cu (Cu, 01-085-1326, orange), cuprite ($Cu_2O$, 01-078-2076, blue), and tenorite (CuO, 01-080-1268, black).

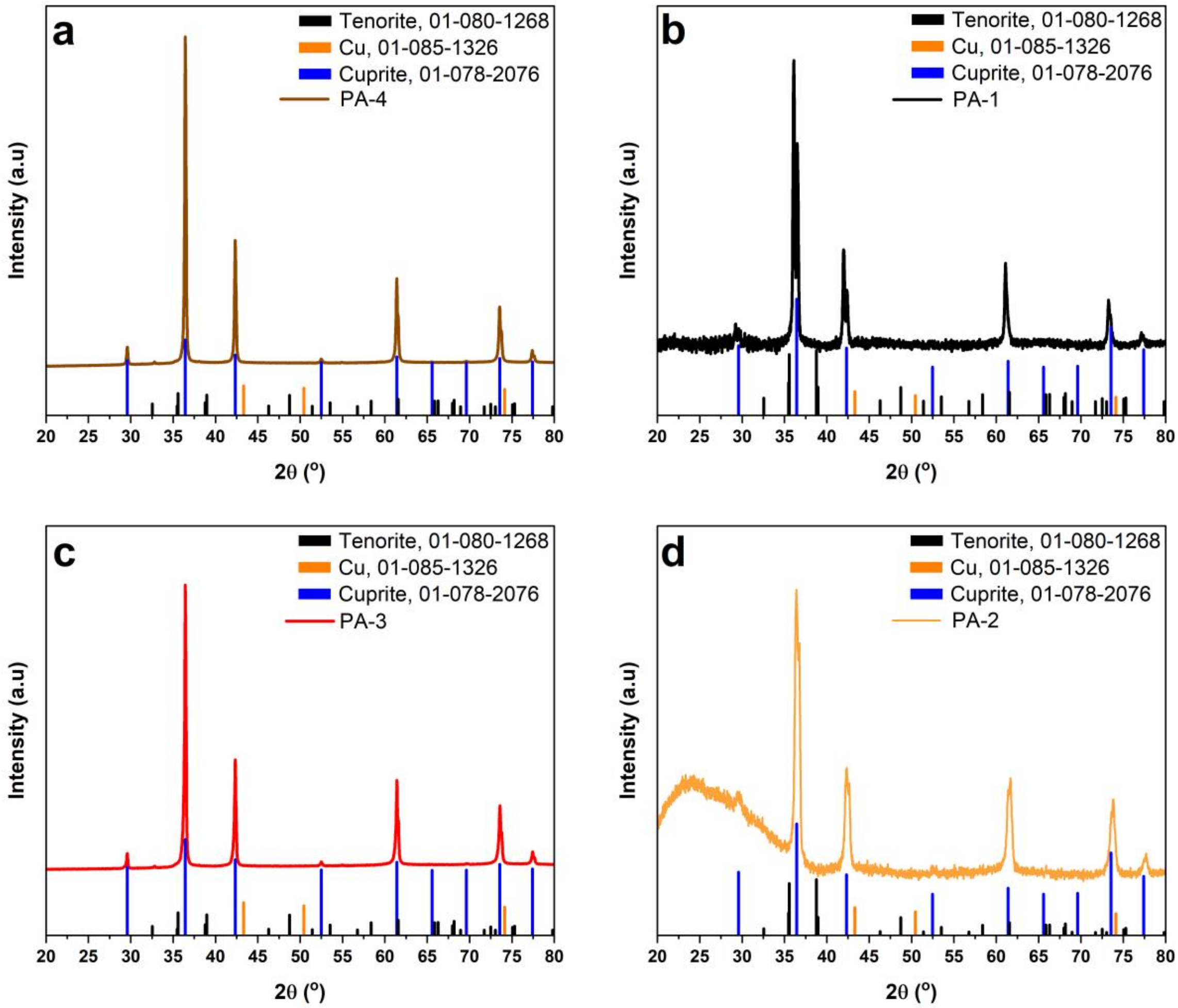


**Figure S8**. XRPD patterns of PA-samples: effect of the initial volume of deionized water, namely: (a) PA-4 (500 mL; brown), (b) PA-1 (400 mL; black), (c) PA-3 (200 mL; red), and (d) PA-2 (100 mL; dark yellow). XRPD reference patterns: metallic Cu (Cu, 01-085-1326, orange), cuprite ($Cu_2O$, 01-078-2076, blue), and tenorite (CuO, 01-080-1268, black).

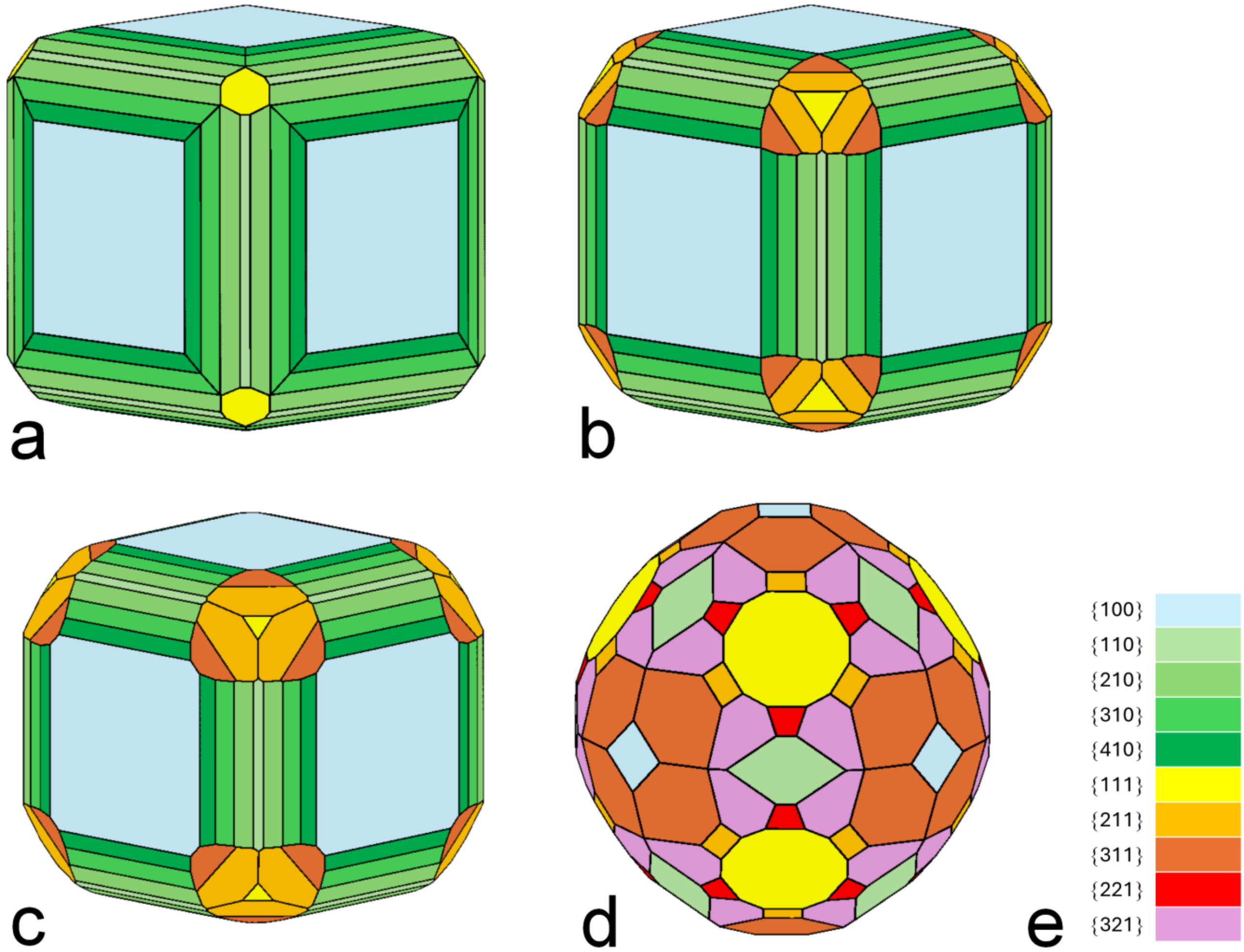


**Figure S9**: The cuprite crystal morphologies reported in the morphodrome of **Figure 11**, namely: (a) a cubic habit with 98 high-index facets (sample PA-4), (b) cubic habit with 146 high-index facets (sample PA-1); (c) cubic habit with 194 high-index facets (sample PA-3), (d) spheroidal crystal with 146 high-index facets (PA-2), (e) explanation of color coding and Miller indexes.

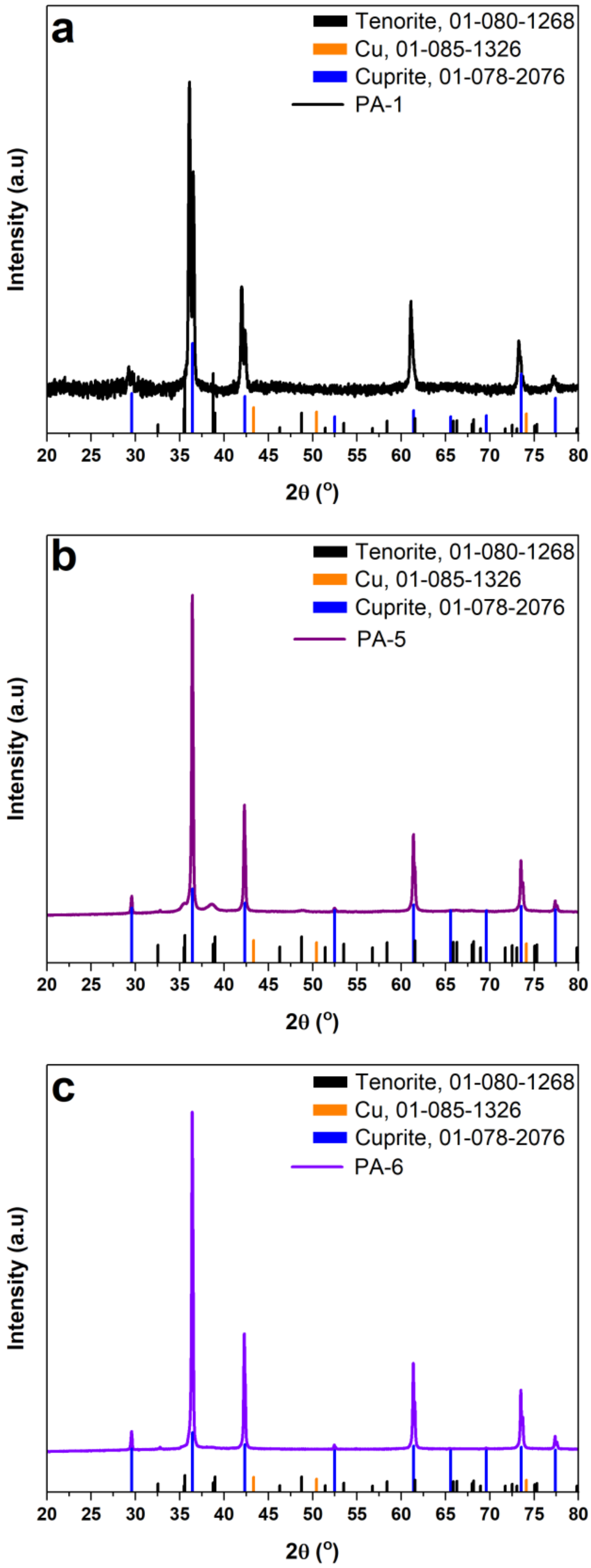


**Figure S10**. XRPD patterns of PA-samples: effect of the reaction temperature, namely: (a) PA-1 (RT; black), (b) PA-5 (50 °C; purple), and (c) PA-6 (75 °C; violet). XRPD reference patterns: metallic Cu (Cu, 01-085-1326, orange), cuprite ($Cu_2O$, 01-078-2076, blue), and tenorite (CuO, 01-080-1268, black).

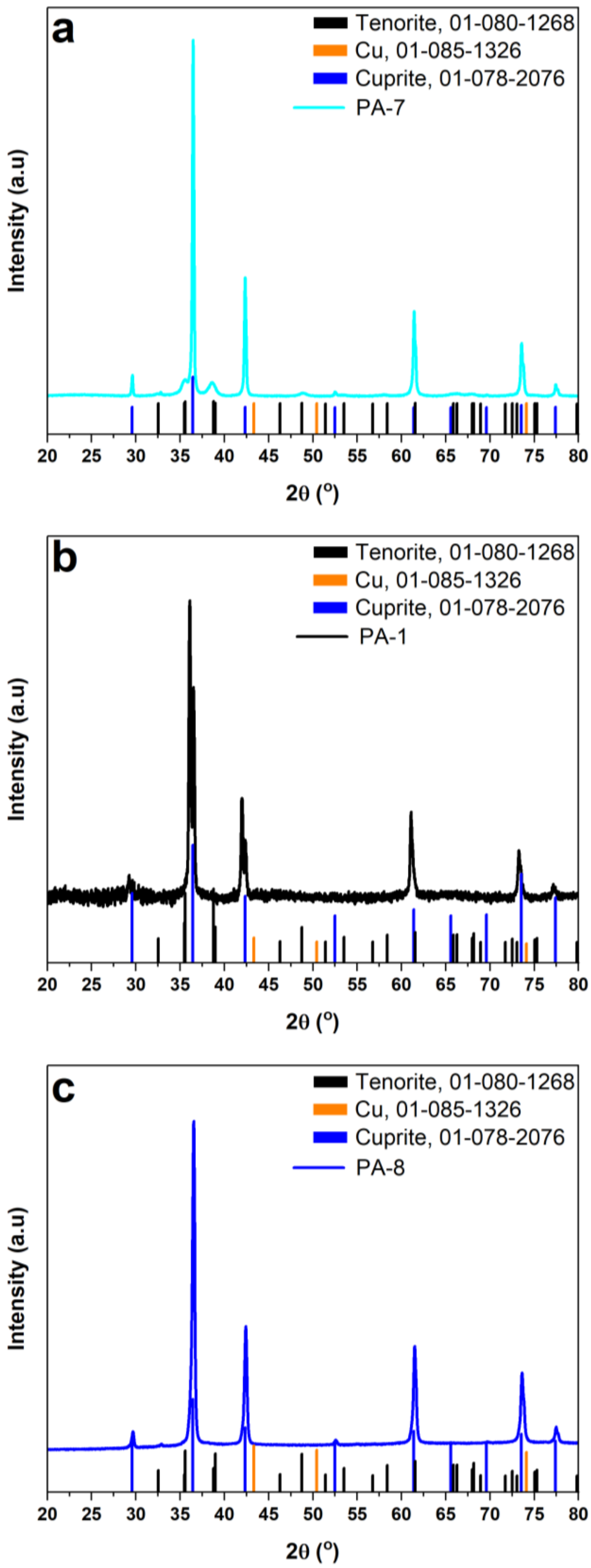


**Figure S11**. XRPD patterns of PA-samples: effect of the Cu(II) salts, and PVP molecular weight, namely: (a) PA-7 (Nitrate, PVP-10k; cyan), (b) PA-1 (Acetate, PVP-10k; black), and (c) PA-8 (Acetate, PVP-55k; blue). XRPD reference patterns: metallic Cu (Cu, 01-085-1326, orange), cuprite ($Cu_2O$, 01-078-2076, blue), and tenorite (CuO, 01-080-1268, black).

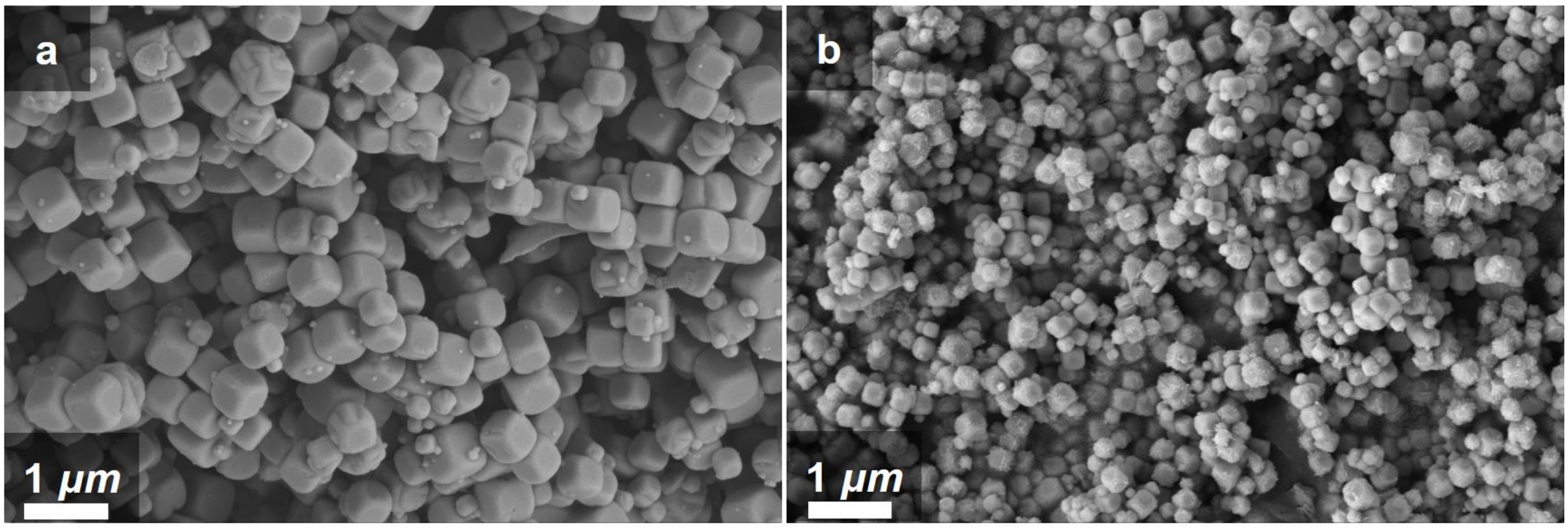


**Figure S12**. SEM micrographs of PA-samples: effect of the time prior to add the AA solution, namely: (a) PA-1 (immediately), and (b) PA-11 (after 10 minutes). All micrographs were collected at the same magnification (30k ×).

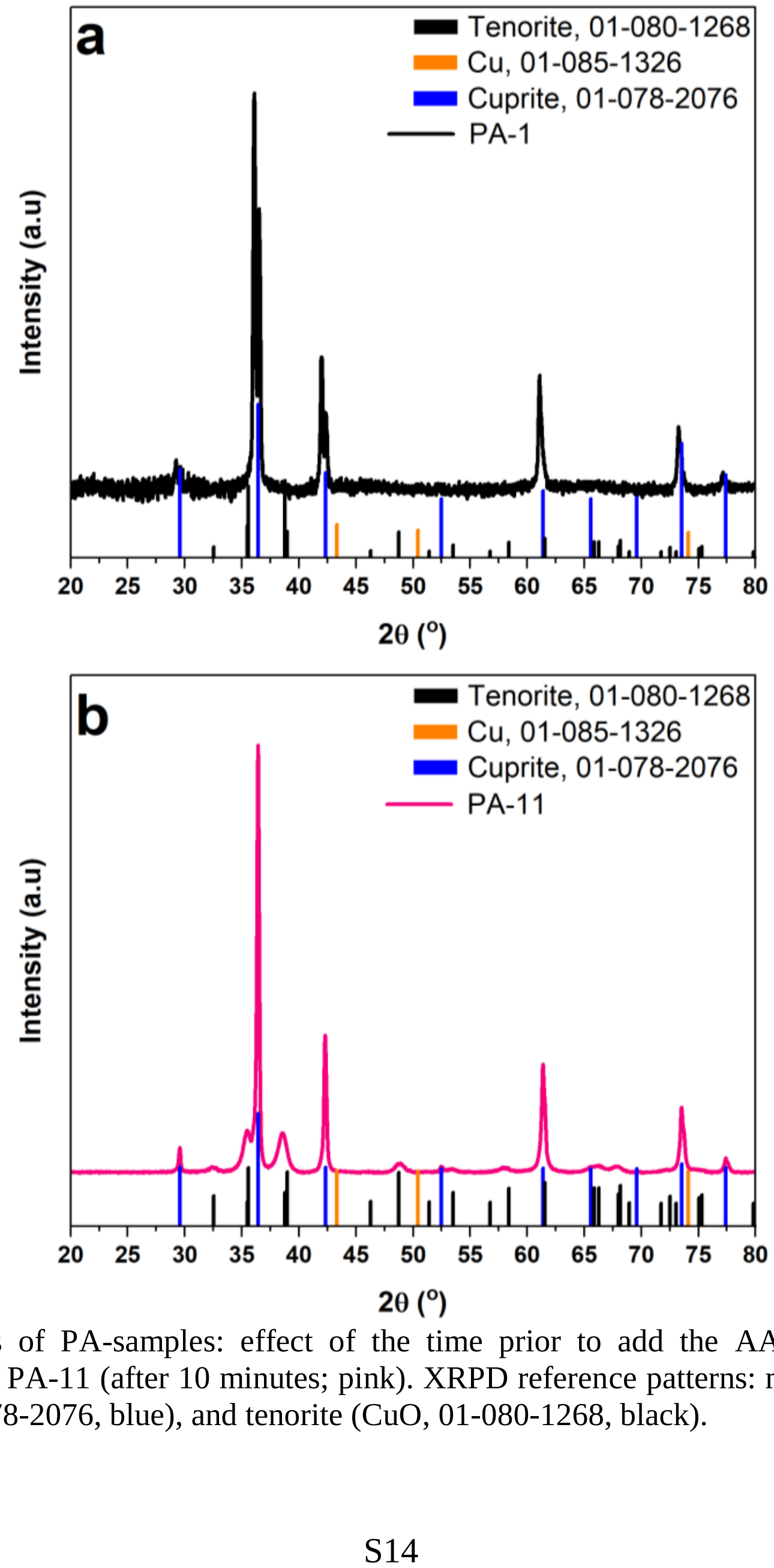


**Figure S13**. XRPD patterns of PA-samples: effect of the time prior to add the AA solution, namely: (a) PA-1 (immediately; black), and (b) PA-11 (after 10 minutes; pink). XRPD reference patterns: metallic Cu (Cu, 01-085-1326, orange), cuprite ($Cu_2O$, 01-078-2076, blue), and tenorite (CuO, 01-080-1268, black).

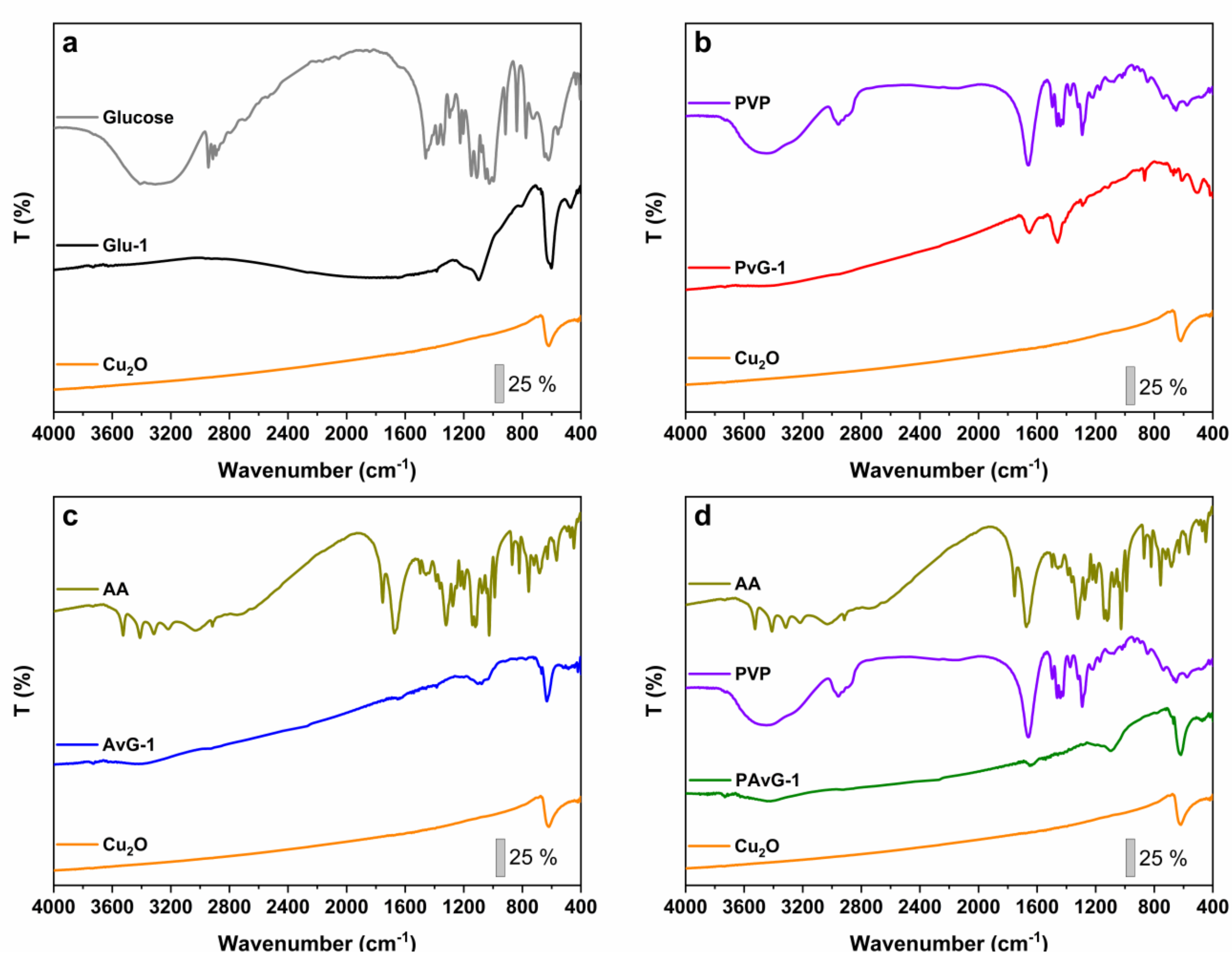


**Figure S14.** FTIR spectra in transmission mode of samples obtained by replacing glucose with PVP/AA: effect of the capping/reducing agent, namely: (a) Glu-1 (Glu 1.8 g; black), (b) PvG-1 (PVP 1.8 g; red), (c) AvG-1 (AA 1.8 g; blue), and (d) PAvG-1 (PVP 0.7 g, AA 1.7 g; green). FTIR reference patterns: $Cu_2O$ (orange), glucose (grey), PVP (violet), and AA (dark yellow).
